# ASIC1a induces synovial inflammation via the Ca^2+^/NFATc3/ RANTES pathway

**DOI:** 10.7150/thno.37200

**Published:** 2020-01-01

**Authors:** Yihao Zhang, Xuewen Qian, Xiaojuan Yang, Ruowen Niu, Sujing Song, Fei Zhu, Chuanjun Zhu, Xiaoqing Peng, Feihu Chen

**Affiliations:** 1 Anhui Key Laboratory of Bioactivity of Natural Products, School of Pharmacy, Anhui Medical University, Hefei 230032, China;; 2 The Key Laboratory of Anti-inflammatory and Immune Medicine, Anhui Medical University, Ministry of Education, Hefei 230032, China.

**Keywords:** ASIC1a, inflammation, rheumatoid arthritis, NFATc3

## Abstract

**Rationale:** Synovial inflammation is one of the main pathological features of rheumatoid arthritis (RA) and is a key factor leading to the progression of RA. Understanding the regulatory mechanism of synovial inflammation is crucial for the treatment of RA. Acid-sensing ion channel 1a (ASIC1a) is an H^+^-gated cation channel that promotes the progression of RA, but the role of ASIC1a in synovial inflammation is unclear. This study aimed to investigate whether ASIC1a is involved in the synovial inflammation and explore the underlying mechanisms *in vitro* and *in vivo*.

**Methods:** The expression of ASIC1a and nuclear factor of activated T cells (NFATs) were analyzed by Western blotting, immunofluorescence, and immunohistochemistry both *in vitro* and *in vivo*. The Ca^2+^ influx mediated by ASIC1a was detected by calcium imaging and flow cytometry. The role of ASIC1a in inflammation was studied in rats with adjuvant-induced arthritis (AA). Inflammatory cytokine profile was analyzed by protein chip in RA synovial fibroblasts (RASF) and verified by a magnetic multi-cytokine assay and ELISA. The NFATc3-regulated *RANTES* (Regulated upon activation, normal T cell expressed and secreted) gene transcription was investigated by ChIP-qPCR and dual-luciferase reporter assay.

**Results:** The expression of ASIC1a was significantly increased in human RA synovial tissues and primary human RASF as well as in ankle synovium of AA rats. Activated ASIC1a mediated Ca^2+^ influx to increase [Ca^2+^]i in RASF. The activation/overexpression of ASIC1a in RASF up-regulated the expression of inflammatory cytokines RANTES, sTNF RI, MIP-1a, IL-8, sTNF RII, and ICAM-1 among which RANTES was increased most remarkably. *In vivo*, ASIC1a promoted inflammation, synovial hyperplasia, articular cartilage, and bone destruction, leading to the progression of AA. Furthermore, activation of ASIC1a upregulated the nuclear translocation of NFATc3, which bound to RANTES promoter and directly regulated gene transcription to enhance RANTES expression.

**Conclusion:** ASIC1a induces synovial inflammation, which leads to the progression of RA. Our study reveals a novel RA inflammation regulatory mechanism and indicates that ASIC1a might be a potential therapeutic target for RA.

## Introduction

Rheumatoid arthritis (RA) is a chronic systemic autoimmune disease that is characterized by joint synovitis 1. It affects approximately 1% of the population worldwide with pathological features including chronic synovitis with inflammatory cell infiltration, non-specific and symmetrical inflammation of the peripheral joints, synovial hyperplasia, and erosion of articular cartilage and bone 3. As the main pathological and pathogenic factor, synovial inflammation plays a crucial role in the occurrence and progression of RA mediating articular cartilage and bone destruction 4-6. However, the pathogenesis of synovial inflammation in RA remains unclear. The primary clinical treatment strategy of RA consists of alleviating the symptoms of inflammation rather than its root causes. Non-steroidal anti-inflammatory drugs (NSAIDs) are used to relieve inflammation and pain, and disease-modifying anti-rheumatic drugs (DMARDs) are employed to delay articular cartilage and bone erosion caused by inflammation 7-9. Although these drugs may help ease the symptoms of RA, joint inflammation continues to develop, leading to joint dysfunction, and eventually, the patients inevitably lose mobility 8. Therefore, the key to the prevention of RA progress is to elucidate the pathogenesis of synovial inflammation and to discover new targets for controlling the development of inflammation.

Extracellular acidification is a common phenomenon that plays an important role in inflammation-associated physiological and pathological processes such as early wound healing, infectious diseases, bone remodeling, and tumorigenesis 10-12. Studies have shown that extracellular acidification induces neutrophil activation to promote inflammation 13, 14. Moreover, extracellular acidification promotes inflammatory cell defense against pathogens by regulating migration and phagocytosis 14, 15. It is noteworthy that extracellular acidification has been shown to be related to the development of RA and the pH of synovial fluid in RA patients is weakly acidic and may drop below 6.0 during active RA 16-19. Furthermore, acidification of synovial fluid is associated with radiological joint destruction in patients with RA 20 and might be a key factor leading to synovial inflammation in the progression of RA. Therefore, it is necessary to elucidate the role of extracellular acidification and its underlying molecular mechanism in synovial inflammation.

Acid-sensing ion channels (ASICs) are a class of extracellular H^+^ activated cationic channels, also known as H^+^ non-voltage-gated cationic channels, which belong to the epithelial sodium channel (ENaC)/degraded protein (DEG) superfamily 21. At present, seven ASIC subunits (ASIC1a, ASIC1b1, ASIC1b2, ASIC2a, ASIC2b, ASIC3, and ASIC4) encoded by four genes (ACCN1, ACCN2, ACCN3, and ACCN4) have been reported 22. As acid receptors on the cell membrane, ASICs transmit the low pH signal of the extracellular microenvironment into the cell to activate downstream signaling pathways and induce a series of physiological and pathological changes 23. Compared with other ASIC subunits, ASIC1a is not only permeable to Na^+^ but also mediates extracellular Ca^2+^ influx 24, 25. Ca^2+^ is an important second messenger that plays a pivotal role in the physiological and pathological processes of cells 26, 27. Our previous studies have reported that ASIC1a promotes acid-induced cartilage destruction by mediating Ca^2+^ influx, which leads to the progression of RA 28-30. Although ASIC1a is an important factor that promotes the progression of inflammation 31-34, it is not known whether ASIC1a is mechanistically involved in synovial inflammation.

NFATs are a group of Ca^2+^-dependent transcription factors that are widely expressed in mammalian cells. To date, five NFAT proteins encoded by five genes have been found in human cells, namely NFATc1 (also known as NFAT2 and NFATc), NFATc2 (also known as NFAT1 and NFATp), NFATc3 (also known as NFAT4 and NFATx), NFATc4 (also known as NFAT3) and NFAT5 (also known as TonEBP and OREBP) 35, 36. Cytoplasmic NFATc1, 2, 3, and 4 are dephosphorylated via extracellular Ca^2+^ influx-mediated calmodulin-calcineurin pathway, which then translocate to the nucleus to regulate gene expression 35, 36. NFAT5 cannot be regulated by Ca^2+^ due to the lack of a calcineurin-binding domain, but it is highly sensitive to salt-induced hypertonicity 37. NFATs act as crucial transcription factors that regulate T cell activation and differentiation and thymocyte development 38, 39. NFATs have been shown to regulate the expression of many inflammatory cytokines such as IL-2, IL-17, GM-CSF, IL-4, and IL-5 involved in the process of inflammation 40-44. Furthermore, it has been reported that NFAT5 promotes macrophage survival to enhance chronic inflammation in RA 45. However, whether NFATs are involved in synovial inflammation by regulating inflammatory cytokines in RA is not known.

We hypothesized that extracellular acidification causes synovial inflammation by activating ASIC1a, which then induces nuclear translocation of NFATs to regulate the expression of inflammatory cytokines. In this study, we investigated the role of ASIC1a in synovial inflammation of RA and found the expression of ASIC1a to be significantly increased both *in vitro* and *in vivo*. Activated ASIC1a mediated Ca^2+^ influx to increase [Ca^2+^]i in RASF. Thus, following overexpression of ASIC1a, the expression of inflammatory cytokines RANTES, sTNF RI, MIP-1a, IL-8, sTNF RII, and ICAM-1 was up-regulated in RASF with the increase of RANTES expression being the most obvious. *In vivo*, ASIC1a promoted inflammation, synovial hyperplasia, and articular cartilage and bone destruction, leading to the progression of adjuvant-induced arthritis (AA) in rats. Furthermore, activation of ASIC1a enhanced nuclear translocation of NFATc3, which regulated transcription of the RANTES gene and enhanced protein expression of RANTES. Thus, our results have elucidated the role of ASIC1a in synovial inflammation, and identified it as a potential therapeutic target to improve the clinical treatment of RA.

## Methods

### Patient enrollment and tissue collection

This study was approved by the Human Research Ethics Committee of Anhui Medical University (China). RA knee synovial tissues were obtained from 22 RA patients (8 men and 14 women) who underwent knee replacement between 2017 and 2018. The mean age of the patients was 61.7±6.8 years. Normal knee synovial tissues were obtained from 3 male patients (aged at 36, 41, and 47) who underwent traffic accident amputation between 2017 and 2018.

### Isolation and culture of synovial fibroblasts

Under sterile conditions, knee synovial tissues were minced into small pieces (about 1 mm^3^). The tissues were aspirated using a Pasteur pipette and evenly attached to the wall of the cell culture flask and cultured in DMEM/high glucose medium (Hyclone, USA) supplemented with 20% FBS (Gibco, USA), 100 IU/mL penicillin and 100 μg/mL streptomycin. The culture flask was placed upright in 37 ℃, 5% CO^2^ cell incubator and then flat after 6 h. After the formation of synovial fibroblast colony, the cells were sub-cultured 3 times. The identified cells were used in subsequent experiments.

### Experimental animals

A total of 120 male Sprague-Dawley rats weighing 120 ± 20 g were provided by the Experimental Animal Center of Anhui Medical University, Hefei, China. The rats were housed in a specific pathogen-free room with controlled ambient temperature (22 ± 2 ℃) and allowed free access to standard pelleted food and water. All experimental protocols described in this study were approved by the Animal Experimental Ethics Review Committee of Anhui Medical University.

### AA induction

The rats received intradermal immunization into the left hind metatarsal footpad with 0.1 mL heat-killed mycobacteria (10 mg/mL) contained in Complete Freund's adjuvant (CFA, Chondrex Inc., USA). The day of CFA injection was designated day 0; the secondary polyarthritis reaction occurred on day 20 in CFA-injected rats.

### Treatment

The specific ASIC1a inhibitor psalmotoxin-1 (PcTx-1, Abcam, UK, 0.5, 1 and 2 μg/kg, once every 3 days, total eight times) was administered by intra-articular injection into each paw of AA rats (day 20, n=8 per group). Positive control rats (day 20, n=8 per group) were injected intra-articularly with triamcinolone acetonide (TA, 1 mg/kg, Selleck, USA, once every 3 days, total eight times). Vehicle rats were injected with normal saline (day 20, n=8 per group). Intra-articular injection was performed with a 31-gauge microliter syringes (Hamilton, Switzerland).

### Arthritis assessment

The arthritic severity in each paw was evaluated by a scoring system that ranges from 0 to 4: 0 = paws with no swelling and focal redness; 1 = paws with swelling of finger joints; 2 = paws with mild swelling of ankle or wrist joints; 3 = paws with severe inflammation of the entire paws; and 4 = paws with deformity or ankylosis. The cumulative score for all four paws of each rat was used as the polyarthritis index, with a maximum value of 16. The right hind paws of rats were measured by toe volume measuring instrument on day 0, 20, 23, 26, 29, 32, 35, 38, and 41.

### Membrane and nuclear protein extract

Cell membrane proteins were extracted from the cell suspension (a total of 5×10^7^ cells) using a membrane protein extraction kit (Beyotime, China). Cell nuclear proteins were extracted from the cell suspension (a total of 2×10^7^ cells) using a nuclear protein extraction kit (KeyGEN, China). Membrane proteins and nuclear proteins were used for further experiments as described below.

### Flow cytometry to identify cells and detect protein expression

Cells were digested and suspended into DMEM/high glucose medium. Next, cells were washed three times for 5 min with phosphate-buffered saline (PBS) and 400 μL 4% paraformaldehyde was added for 15 min. After washing three times with PBS, the blocking solution was added for 30 min. Subsequently, the washing step was repeated, and the anti-CD55 (Santa Cruz, SC-21769, USA) or anti-ASIC1a antibodies (Ptoteintech, 27235-1-AP, China) were added and kept at 4 ℃ in the dark overnight. The next day, cells were washed three times for 5 min with PBS, 500 μL fluorescent secondary antibody solution was added for 1h at room temperature, and the washing step was repeated. Samples were detected by a flow cytometer (Beckman Coulter, USA), and results were quantitatively analyzed by the Kaluza Analysis Software (Beckman Coulter, USA) to determine the mean fluorescence intensity.

### Silencing of ASIC1a or NFATc3

Transfection was performed in a six-well plate. RASF transfected with 0.5 mM ASIC1a or NFATc3-specific shRNA lentivirus particles (Genechem, China) for 10 h in 1 mL of complete medium with 5 mg/mL polybrene per well; other wells were transfected with control shRNA lentivirus particles. The medium in each well was replaced with 1 mL of complete medium (without Polybrene), and 3 mg/mL purinomycin dihydrochloride was used to screen stable clones expressing shRNA. One week later, stable colonies were expanded for further study. The shRNA target sequences of ASIC1a or NFATc3 are shown in Table S1.

### Overexpression of ASIC1a

Transfection was performed in a six-well plate. RASF were transfected with 0.5 mM *ASIC1a* gene lentivirus particles (Genechem, China) for 10 h in 1 mL of complete medium with 5 mg/mL polybrene per well; other wells were transfected with empty plasmid lentivirus particles. The medium in each well was replaced with 1 mL of complete medium (without Polybrene), and 3 mg/mL purinomycin dihydrochloride was used to screen stable clones expressing the gene. One week later, stable colonies were expanded for further study. The primer for ASIC1a overexpression is shown in Table S1.

### Western blotting

Cultured cells were lysed with RIPA lysis buffer containing 1% protease inhibitor cocktail (Beyotime, China). The protein concentration in the lysates was measured using a BCA protein assay kit (Beyotime, China). Protein samples were separated by 10% SDS-polyacrylamide gel and then transferred to polyvinyl difluoride membranes (Millipore, USA), which were then blocked for 1 h with 5% skim milk in TBST (10 mM Tris, 150 mM NaCl, and 0.05% Tween 20 (pH8.3)) at room temperature. The membranes were incubated overnight at 4 ℃ with anti-ASIC1a antibody (Ptoteintech, 27235-1-AP, China) or anti-NFATc2(Abcam, ab92490, UK), anti-NFATc1 (Abcam, ab177464, UK), anti-NFATc3 (Abcam, ab93628, UK), anti-NFAT5 (Abcam, ab137407, UK), anti-NFATc4 (CST, 2188, USA), anti-Na^+^/K^+^-ATPase (Abcam, ab76020, UK), anti-H3 (Abcam, ab1791, UK), anti-β-actin (ZSGB Bio, TA-09, China) antibodies. The membranes were washed in TBST and incubated with secondary antibody (1:5000, ZSGB Bio, China) for 1 h at room temperature followed by exposure to electrochemiluminescence. The results were expressed as a percentage of control signals in each blot to correct for variations between blots.

### Immunohistochemistry and hematoxylin-eosin (HE) staining

The rat right hind ankle joint was soaked in EDTA decalcifying solution for two months. Immunohistochemistry (IHC) staining was performed according to the protocol in the SP9000 IHC reagents kit (ZSGB Bio, China), and HE staining was performed according to the protocol in the HE staining kit (Beyotime, China). Each sample was observed by a digital pathology slide scanner (3DHISTECH, Hungary). The IHC results were quantitatively analyzed by the Image-ProPlus Software (MEDIA CYBERNETICS, USA) to calculate the integral optical density (IOD).

### Immunofluorescence staining

Sections from paraffin-embedded joints were deparaffinized with xylene and rehydrated with graded alcohols. Immunofluorescence staining was performed by incubating cells with anti-ASIC1a or anti-NFATc2, anti-NFATc1, anti-NFATc4, anti-NFATc3, anti-NFAT5 (Bioss, China) antibodies in glass-bottom dishes according to our previously described method 46. For nuclear staining, cells were incubated with DAPI. Samples were imaged using a confocal microscope (Zeiss, Germany).

### Enzyme-linked immunosorbent assay (ELISA)

Macrophage inflammatory protein-1a (MIP-1a) in cell supernatants was quantified using the human MIP-1a ELISA kit (RayBiotech, USA) according to the manufacturer's protocol. This assay employed an antibody specific for human MIP-1a coated on a 96-well plate. Standards and samples were pipetted into the wells, and MIP-1a present in the sample was bound to the wells by the immobilized antibody. The wells were washed, and biotinylated anti-human MIP-1a antibody was added. After washing away unbound biotinylated antibody, HRP-conjugated streptavidin was pipetted to the wells which were again washed. TMB substrate solution was added to the wells allowing color development in proportion to the amount of MIP-1a bound. The Stop Solution changed the color from blue to yellow, and the intensity of the color was measured at 450 nm.

### Inflammatory cytokines antibody array

Cells were treated for 6 h in DMEM/high glucose medium containing 1% FBS. After 6 h, the cell supernatant of each treatment group was collected. Inflammatory cytokines in cell supernatant were quantified using the human inflammation antibody array kit (RayBiotech, AAH-INF-G3, USA) according to the manufacturer's protocol. Briefly, after adjusting the protein glass chips into the incubation chamber, the chips were blocked by adding 100 μL 1× blocking buffer to each well for 30 min at room temperature. Then, the blocking buffer was discarded and 100 μL of cell supernatants were added to the wells of the protein chip and incubated overnight at room temperature. Supernatants were discarded, and the chips were washed five times for 2 min with 150 μL wash buffer I and twice for 2 min with 150 μL wash buffer II. Thereafter, 70 μL of biotin-conjugated antibody solution was added to each well of the protein chip and incubated for 2 h at room temperature. The chips were again washed with wash buffers I and II. Next, 70 μL of 1500× Alexa Fluor 555-conjugated streptavidin solution was added to each well and incubated overnight at 4 ℃ in the dark. After repeating the cleaning step, glass chips were dried, and fluorescence was quantified at 532 nm using an InnoScan300 microarray scanner (Innopsys, France). Distribution of fluorescence arrays of 40 inflammatory cytokines is presented in Table S2.

### Calcium Imaging

Cells on a glass dish were washed three times with D-Hanks' solution and incubated with 5 μM Fluo 3-AM (Dojindo Laboratories, Japan) for 30 min at 37℃, followed by three washes and additional incubation in normal Hanks' solution for 15 min. To eliminate the effects of other Ca^2+^ channels or intracellular Ca^2+^ stores release, 1 mM extracellular calcium chelator EGTA, 10 μM intracellular calcium chelator BAPTA-AM (Cells were first treated with 10 uM BAPTA-AM in DMEM/high glucose medium without FBS for 30 min, then the subsequent experiments were performed), 5 μM L-type calcium channel blocker verapamil (Sigma, USA) and 5 μM selective Na^+^/H^+^ exchanger inhibitor 5-(N, N-Hexamethylene) amiloride (HMA, Alomone Labs, Israel) were added to the extracellular fluid.Using a laser scanning confocal microscope, Fluo 3-AM was excited at 488 nm, and emission was measured at 510 nm.

### Detection of [Ca^2+^]i using flow cytometry

Cell suspensions in Eppendorf tubes were washed three times with D-Hanks' solution and incubated with 5 μM Fluo 3-AM (Dojindo Laboratories, Japan) for 30 min at 37 ℃. Next, cells were washed three times for 5 min and were incubated in normal Hanks' solution for 15 min. Using a flow cytometer, Fluo 3-AM was excited at 488 nm, and emission was measured at 510 nm.

### Magnetic multi-cytokine assay

Cells were treated for 6 h in DMEM/high glucose medium containing 1% FBS. After 6 h, the cell supernatant of each treatment group was collected. Inflammatory cytokines RANTES, sTNF RI, IL-8, sTNF RII, and ICAM-1 in cell supernatants were quantified using the Magnetic Luminex Assay multiplex kits (R&D Systems, LXSAHM-05, USA) according to the manufacturer's protocol. The samples were read within 90 min using a Luminex X-200 analyzer (Luminex, USA).

### Chromatin immunoprecipitation-quantitative real-time PCR (ChIP-qPCR)

The human RANTES promoter sequence (3500 bp) was predicted using the UCSC genome bioinformatics program (http://genome.ucsc.edu/index.html) and the putative binding sites for NFATc3 in the RANTES gene promoter were predicted using the JASPAR database (http://jaspar.genereg.net/). Binding of NFATc3 to the RANTES promoter was examined by chromatin immunoprecipitation (ChIP) assay in RASF. In brief, cells cultured in 10cm dishes were cross-linked with 1% formaldehyde for 10 min at room temperature, and the reaction was stopped with glycine. The genomic DNA was then sheared into fragments with an average size of 200-500 bp. Subsequently, immunoprecipitation was performed with anti-NFATc3, and immunoprecipitation with nonspecific IgG was used as a negative control. Quantitative real-time PCR (qPCR) was performed to detect DNA fragments of the RANTES promoter region. The designed three primers used to amplify NFATc3-binding elements are listed in Table S3. Fluorescent signals were collected during the extension phase, Ct values of the sample were calculated, and transcript levels were analyzed by the 2^-ΔCt^ method.

### Dual-luciferase reporter assay

We amplified gene regions upstream of the RANTES promoter 1812 bp relative to the transcriptional start site by PCR to generate RANTES promoter constructs (Table S3). RASF were co-transfected with RANTES promoter firefly luciferase and NFATc3 expression plasmids using X-tremegene HP reagent (ROCHE, Switzerland). Twenty-four h after transfection, luciferase activity was measured using the Dual-Luciferase Reporter Assay System (Promega, E1910, USA) according to the manufacturer's protocol and normalized to Renilla luciferase activity.

### Statistical analysis

Each experiment was performed at least in triplicate, and the measurements were performed in three independent experiments. The data are expressed as mean ± SEM. Student's t-test was used to compare the differences between means of two groups, and one-way ANOVA was used to compare the means of three or more groups to determine whether they differ significantly from one another. *P*<0.05 was considered statistically significant (*P<0.05,**P<0.01, ***P<0.001). All analyses were performed using the GraphPad Prism 6 software (GraphPad Software, USA).

## Results

### ASIC1a is highly expressed in human RA synovial tissues and primary human RASF

**Figure 1:**
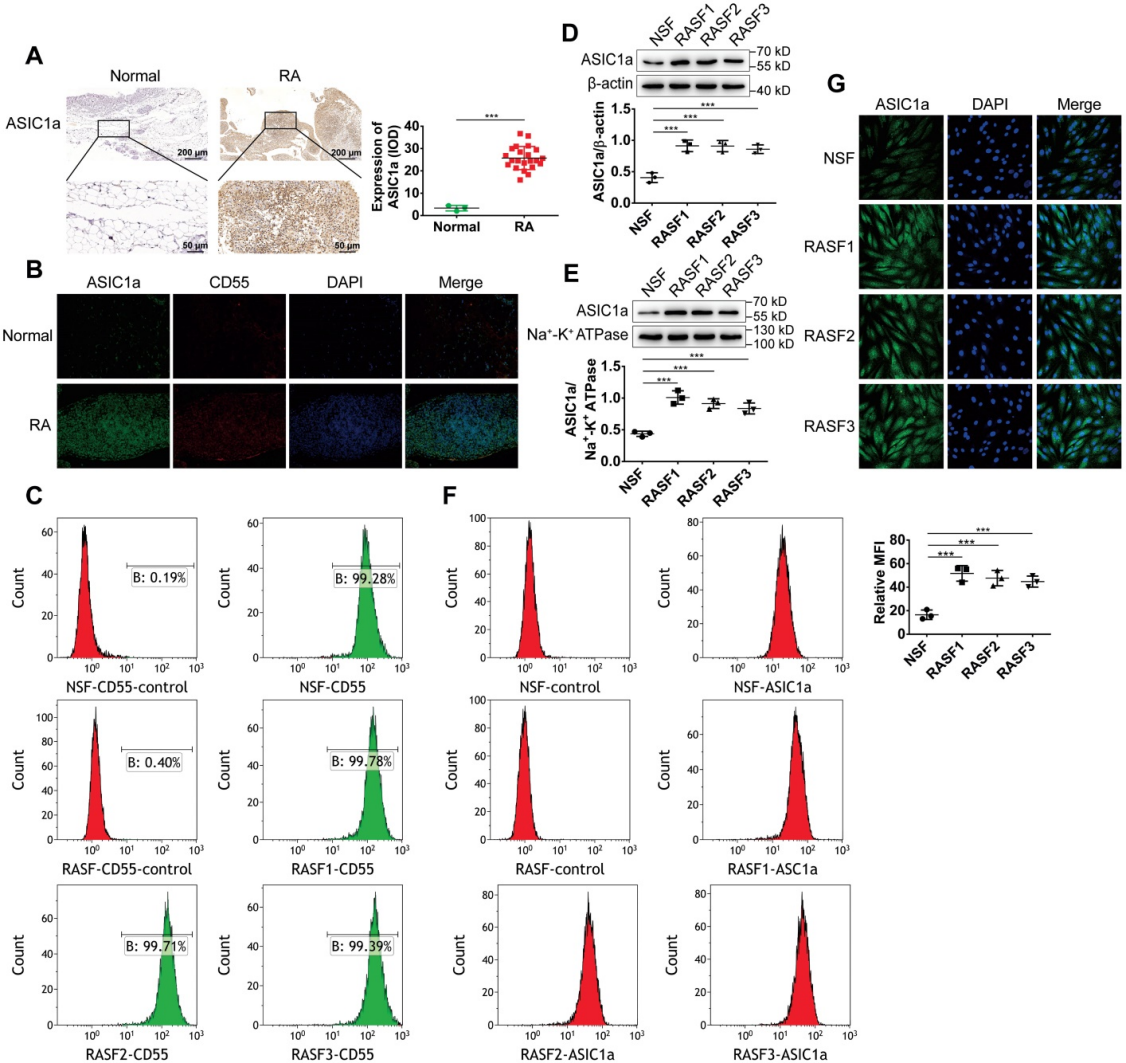
** ASIC1a is highly expressed in human RA synovial tissues and primary human RASF. (A)** Immunohistochemistry analysis of ASIC1a expression in human normal and RA synovial tissues. **(B)** Immunofluorescence analysis of ASIC1a expression in human normal and RA synovial tissues. The scale bars are 100 μm. **(C)** Cells identified by flow cytometry. **(D)** Western blot analysis of total ASIC1a expression in NSF and RASF. **(E)** Western blot analysis of membrane ASIC1a expression in NSF and RASF. **(F)** Membrane ASIC1a expression detected by flow cytometry. MFI, is mean fluorescence intensity. **(G)** Immunofluorescence analysis of ASIC1a expression in NSF and RASF. The scale bars are 20 μm. Student´s t-test or one-way ANOVA was used for statistical analysis, and data are expressed as mean ± SEM for three separate experiments.

We have previously reported that ASIC1a as an acid receptor on the cell membrane transmits the signal of low pH of the extracellular microenvironment into the cell and activate intracellular signaling pathways producing a series of pathological changes in RA 28-30. However, it was not known whether ASIC1a was involved in synovial inflammation. Therefore, we investigated the expression level of ASIC1a in human RA synovial and normal synovial tissues. As shown in Figure 1A-B, the expression levels of the ASIC1a protein were significantly higher in RA synovial tissues than in normal synovial tissues. CD55 acts as a specific surface antigen on synovial fibroblasts that distinguishes synovial macrophages. ASIC1a had a co-expression region with CD55 in the synovial tissue indicating that ASIC1a was highly expressed in synovial fibroblasts of RA synovial tissues.

Next, normal synovial fibroblasts (NSF) from normal synovial tissue of one donor and three RASF from RA synovial tissues of three donors were isolated and cultured. As displayed in Figure 1C, flow cytometry using CD55 as a marker showed that NSF and three RASF were successfully isolated. We further detected the expression level of ASIC1a in RASF and NSF; compared with NSF, the total ASIC1a expression was significantly upregulated in RASF (Figures 1D and 1G). ASIC1a is an H^+^-gated cation channel that functions only when it exists on the membrane. Therefore, it was necessary to detect the expression of ASIC1a on the cell membrane. Western blotting and flow cytometry showed that membrane ASIC1a was highly expressed on RASF compared with NSF (Figure 1E-F). Taken together, these data showed that ASIC1a might be a causally involved in RA.

### ASIC1a has an ion channel activity that mediates Ca^2+^ influx

**Figure 2:**
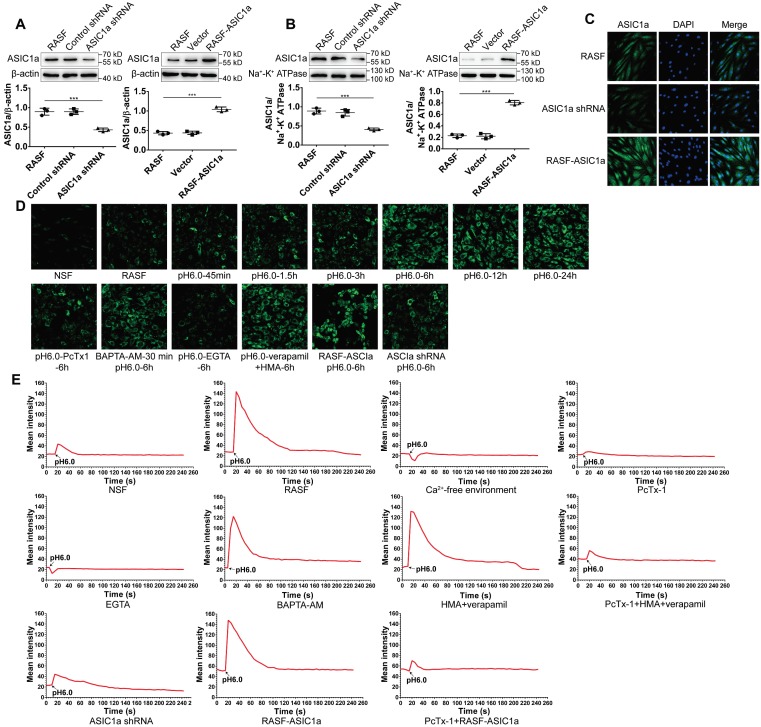
** ASIC1a has an ion channel activity that mediates Ca^2+^ influx. (A)** Total ASIC1a protein was detected by Western blotting in RASF-ASIC1a transfectants and ASIC1a shRNA transfectants. **(B)** Membrane ASIC1a protein was detected by Western blotting in RASF-ASIC1a transfectants and ASIC1a shRNA transfectants. **(C)** Immunofluorescence analysis of ASIC1a expression in RASF-ASIC1a transfectants and ASIC1a shRNA transfectants. The scale bars are 20 μm. **(D)** Calcium imaging detected the effect of ASIC1a on [Ca^2+^]i in RASF with 100 nM PcTx-1, 1 mM EGTA, 10 μM BAPTA-AM, 5 μM verapamil and HMA **(E)** Calcium imaging detected ASIC1a-mediated Ca^2+^ influx in RASF with 100 nM PcTx-1, 1 mM EGTA, 10 μM BAPTA-AM, 5 μM verapamil and HMA. Student´s t-test or one-way ANOVA was used for statistical analysis, and data are expressed as mean ± SEM for three separate experiments.

Compared with other ASICs members, ASIC1a is not only permeable to Na^+^, but also mediates extracellular Ca^2+^ influx. Ca^2+^ is an important second messenger that plays a pivotal role in the physiological and pathological processes of cells, including inflammation 47, 48. Therefore, it was important to determine whether ASIC1a has ion channel activity that mediates Ca^2+^ influx in RASF. First, ASIC1a was overexpressed or silenced by lentiviruses. Western blotting and immunofluorescence staining showed that compared to controls, the total ASIC1a expression was significantly increased or decreased in RASF-ASIC1a or ASIC1a-shRNA transfectants, respectively (Figures 2A and 2C). The expression of membrane ASIC1a also showed similar results (Figures 2B and S1A). The effect of activation of ASIC1a on [Ca^2+^]i in acidic solution (pH 6.0) was investigated by flow cytometry and fluorescent Ca^2+^-imaging. As is evident from Figures 2D and S1B, [Ca^2+^]i was significantly elevated in RASF compared with NSF. [Ca^2+^]i gradually increased in RASF treated with an acid solution for 45 min, 1.5 h, 3 h, 6 h, 12 h, and 24 h and stabilized after 6 h. Additionally, when RASF were treated with 100 nM PcTx-1 in the acid solution for 6 h, the increased level of [Ca^2+^]i was weakened. Similar results were obtained when ASIC1a was silenced, and overexpression of ASIC1a produced the opposite effect (Figures 2D and S1C). Next, 1 mM extracellular calcium chelator EGTA, 10 μM intracellular calcium chelator BAPTA-AM, 5 μM verapamil (an L-type calcium channel blocker) and HMA (a selective Na^+^/H^+^ exchanger inhibitor) were used to demonstrate that the increase of [Ca^2+^]i was mediated by ASIC1a in RASF rather than by intracellular Ca^2+^ stores release or other calcium channels. Figures 2D and S1C show that [Ca^2+^]i was significantly decreased in RASF treated with EGTA compared to H^+^-6 h group, but was not significantly changed by BAPTA-AM or verapamil and HMA. These results indicated that activation of ASIC1a led to increased [Ca^2+^]i in RASF.

Further, we investigated whether ASIC1a mediated Ca^2+^ influx in RASF by fluorescent Ca^2+^-imaging. As shown in Figure 2E, the [Ca^2+^]i was significantly increased in RASF treated with acidic (pH6.0) Ca^2+^-containing solution rather than acidic Ca^2+^-free solution. The acid-induced increase in [Ca^2+^]i was significantly attenuated by PcTx-1 or EGTA; similar results were obtained when ASIC1a was silenced. Verapamil and HMA or BAPTA-AM did not affect the acid-induced increase in [Ca^2+^]i. However, in the presence of PcTx-1, verapamil and HMA, the acid-induced increase in [Ca^2+^]i was diminished. Acid induced the influx of Ca^2+^ in RASF-ASIC1a transfectants that was inhibited after adding PcTx-1. These data demonstrated an essential role for ASIC1a in mediating acid-induced Ca^2+^ influx in RASF.

### ASIC1a upregulates the expression of inflammatory cytokines by mediating Ca^2+^ influx

**Figure 3:**
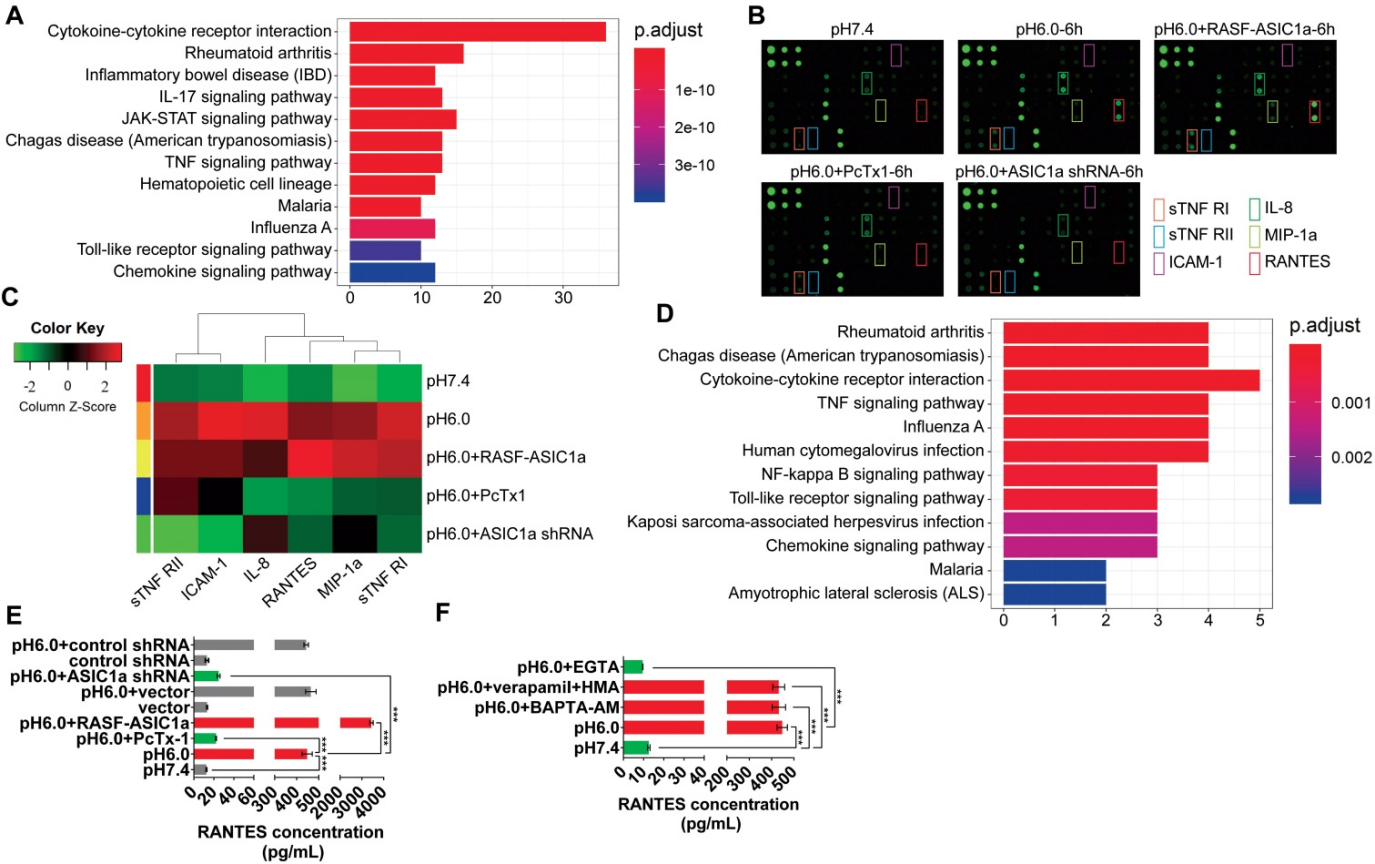
** ASIC1a upregulates the expression of inflammatory cytokines by mediating Ca^2+^ influx. (A)** 40 inflammatory cytokines were analyzed by KEGG pathway enrichment analysis. **(B)** An inflammatory cytokines antibody array was used to examine the expression of 40 inflammatory cytokines in RASF with 100 nM PcTx-1. **(C)** Semi-quantitative analysis of the expressions of RANTES, sTNF RI, MIP-1a, IL-8, sTNF RII, and ICAM-1 by fluorescence intensity presented by heat map. **(D)** RANTES, sTNF RI, MIP-1a, IL-8, sTNF RII, and ICAM-1 were analyzed by KEGG pathway enrichment analysis. **(E)** The expression of RANTES was determined by magnetic multi-cytokine assay. Cells were treated for 6 h with100 nM PcTx-1. **(F)** Magnetic multi-cytokine assay detected the increase of RANTES expression mediated by ASIC1a in RASF treated for 6 h with 1 mM EGTA, 10 μM BAPTA-AM, 5 μM verapamil and HMA. Student´s t-test or one-way ANOVA was used for statistical analysis, and data are expressed as mean ± SEM for three separate experiments.

Previous studies have shown that ASIC1a is an important factor that promotes the development of inflammation 31-34. However, it is not known whether ASIC1a is mechanistically involved in synovial inflammation. Therefore, we used an inflammatory cytokines antibody array to examine the expression of 40 inflammatory cytokines (Table S4). Gene Ontology (GO) enrichment and KEGG pathway enrichment analyses showed that 40 inflammatory cytokines play an important role in inflammation (Figures S2A and 3A). The fluorescence arrays of 40 inflammatory cytokines are displayed and their semi-quantitative analysis is presented by heat map (Figures 3B and S2B). Compared with the pH 7.4 group, RANTES, sTNF RI, MIP-1a, IL-8, sTNF RII, and ICAM-1 expressions in pH 6.0 group and pH 6.0+RASF-ASIC1a group were significantly increased (fold change>1.5). As shown in Figure 3B-C, RANTES was the most significantly affected cytokine with 89.4-fold change in the pH 6.0+RASF-ASIC1a group and 20.9-fold change in the pH 6.0 group. In contrast, the fold change of 6 inflammatory cytokine expressions was weak in pH 6.0+PcTx-1 group and pH 6.0+ASIC1a shRNA group. Furthermore, GO and KEGG pathway enrichment analyses showed that RANTES, sTNF RI, MIP-1a, IL-8, sTNF RII, and ICAM-1 were important inflammatory cytokines for RA (Figures 3D and S2C).

To further confirm the above findings, the expressions of RANTES, sTNF RI, MIP-1a, IL-8, sTNF RII, and ICAM-1 were determined by magnetic multi-cytokine assay and ELISA. As shown in Figure 3E, compared with the pH 7.4 group, increased RANTES expression was most significant in pH 6.0 group and pH 6.0+RASF-ASIC1a group, and the change in RANTES expression was significantly attenuated in pH 6.0+PcTx-1 group and pH 6.0+ASIC1a shRNA groups. Moreover, there was no significant change in RANTES expression in vector and control shRNA groups that were the control transfectants (Figure 3E). A similar trend was found in the expressions of IL-8, MIP-1a, ICAM-1, sTNF RI, and sTNF RII, and the level of increase was evidently weaker than that of RANTES (Figure S2D).

We investigated whether ASIC1a regulated the expression of 6 inflammatory cytokines by mediating Ca^2+^ influx. Our results demonstrated that acid-induced activation of ASIC1a significantly enhanced RANTES expression by mediating Ca^2+^ influx in RASF. ASIC1a also promoted the expressions of IL-8, MIP-1a, ICAM-1, sTNF RI and sTNF RII, but this effect was considerably weaker than that of RANTES. The magnetic multi-cytokine assay showed that acid-induced increase of RANTES expression was significantly attenuated by EGTA compared with the pH 6.0 group, but verapamil and HMA or BAPTA-AM did not affect the increase in RANTES expression (Figure 3F). There was a similar but weaker trend in the expressions of IL-8, MIP-1a, ICAM-1, sTNF RI, and sTNF RII compared to RANTES (Figure S2E).

### ASIC1a promotes inflammation by mediating the expression of inflammatory cytokines *in vivo*

**Figure 4:**
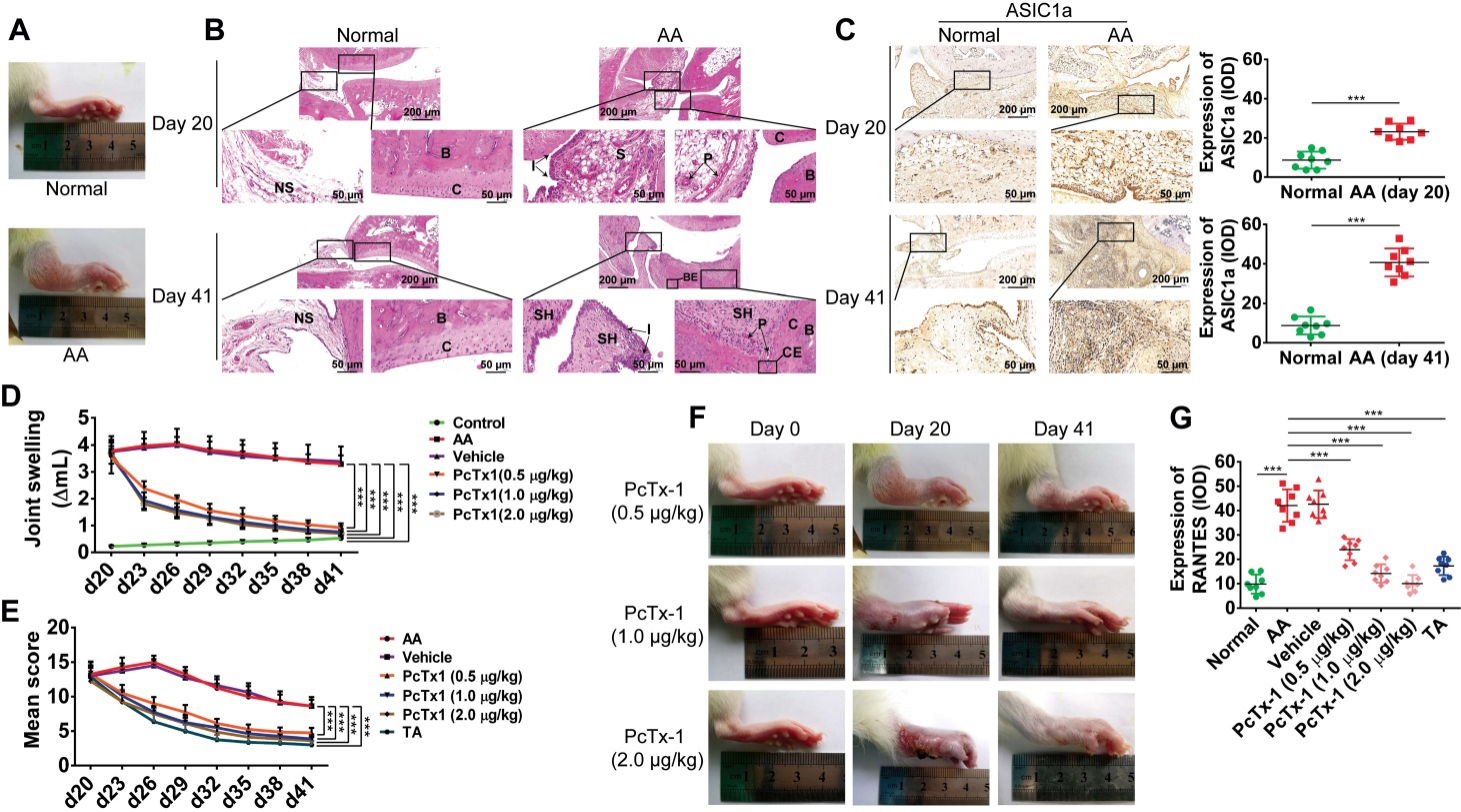
** ASIC1a promotes inflammation by mediating the expression of inflammatory cytokines *in vivo*. (A)** Representative image of right hind paw edema in rats with adjuvant-induced arthritis (AA) on day 20. **(B)** Normal and AA rat right hind ankle joint sections stained with hematoxylin and eosin. Histological section showing subchondral bone (B), bone erosion (BE), articular cartilage (C), cartilage erosion (CE), inflammatory cell infiltration (I), normal synovium (NS), pannus formation (P), synovium (S), and synovial hyperplasia (SH). **(C)** Immunohistochemistry analysis of ASIC1a expression in rat ankle synovium (n = 8). **(D)** The right hind paws of rats were measured by toe volume measuring instrument on day 0, 20, 23, 26, 29, 32, 35, 38, and 41. **(E)** Rat arthritic severity in each paw was evaluated by using a scoring system. **(F)** Representative image of right hind paw edema in rats treated with 0.5, 1, and 2 μg/kg PcTx-1. **(G)** Semi-quantitative analysis of RANTES expression in rat ankle synovium by integral optical density (IOD). Student´s t-test or one-way ANOVA was used for statistical analysis, and data are expressed as mean ± SEM for three separate experiments.

AA is an experimental model of polyarthritis in rats, which has been widely used to study the pathogenesis of RA and preclinical testing of numerous anti-arthritic agents 49, 50. We used the AA rat model to investigate whether ASIC1a could regulate the inflammation of RA *in vivo*. AA rats developed severe arthritis on the 20th day after immunization. As shown in Figure 4A, compared with normal rats, the paws of AA rats showed swelling. HE staining showed pannus formation, and inflammatory cell infiltration appeared in the synovial cavity of AA rats on day 20, and cartilage and bone erosion, as well as synovial hyperplasia, further appeared on day 41 (Figure 4B). These results indicated that the AA rat models were successfully induced. Immunohistochemical analysis showed that the expression of ASIC1a was significantly increased in the synovial membrane of AA rats compared with normal rats (Figure 4C). The specific ASIC1a inhibitor PcTx-1 (0.5, 1 and 2 μg/kg, once every 3 days, total eight times) was administered by intra-articular injection into each paw of AA rats. Compared with the AA model group, the paw swelling of rats was significantly alleviated in 0.5, 1, and 2 μg/kg PcTx-1-treated groups in a dose-dependent manner (Figure 4D and 4F). HE staining showed that synovial hyperplasia, bone erosion, and inflammatory cell infiltration were alleviated, and pannus was reduced in 0.5, 1, and 2 μg/kg PcTx-1-treated groups compared with the AA model group (Figure S3A). Also, the arthritic severity was significantly alleviated in 0.5, 1, and 2 μg/kg PcTx-1 groups compared with the AA model group (Figure 4E). These results suggested that ASIC1a promoted joint inflammation *in vivo*.

We further investigated whether ASIC1a mediated the *in vivo* expression of RANTES, IL-8, MIP-1a, ICAM-1, TNF RI, and TNF RII. Immunohistochemical results showed that the expression of RANTES was increased in the synovial membrane of AA rats compared with normal rats and were significantly decreased in 0.5, 1, and 2 μg/kg PcTx-1-treated groups compared with the AA model group (Figures 4G and S3B). Furthermore, there was a similar but weaker trend in the expressions of IL-8, MIP-1a, ICAM-1, TNF RI, and TNF RII, compared to RANTES (Figures S3B-C). These results showed that ASIC1a mediated RANTES expression *in vivo*. Although ASIC1a promoted the expression of IL-8, MIP-1a, ICAM-1, TNF RI, and TNF RII, this effect was weaker than that of RANTES.

### ASIC1a induces the nuclear translocation of NFATc3 by mediating Ca^2+^ influx

The above results have shown that ASIC1a induced synovial inflammation, but the underlying molecular mechanism remained to be clarified. NFATs are a group of Ca^2+^-dependent transcription factors that play a vital role in inflammation 40-45. However, whether NFATs are involved in synovial inflammation in RA via the regulation of inflammatory cytokines is unknown. Therefore, we explored whether ASIC1a induced nuclear translocation of NFATs by mediating Ca^2+^ influx.

**Figure 5:**
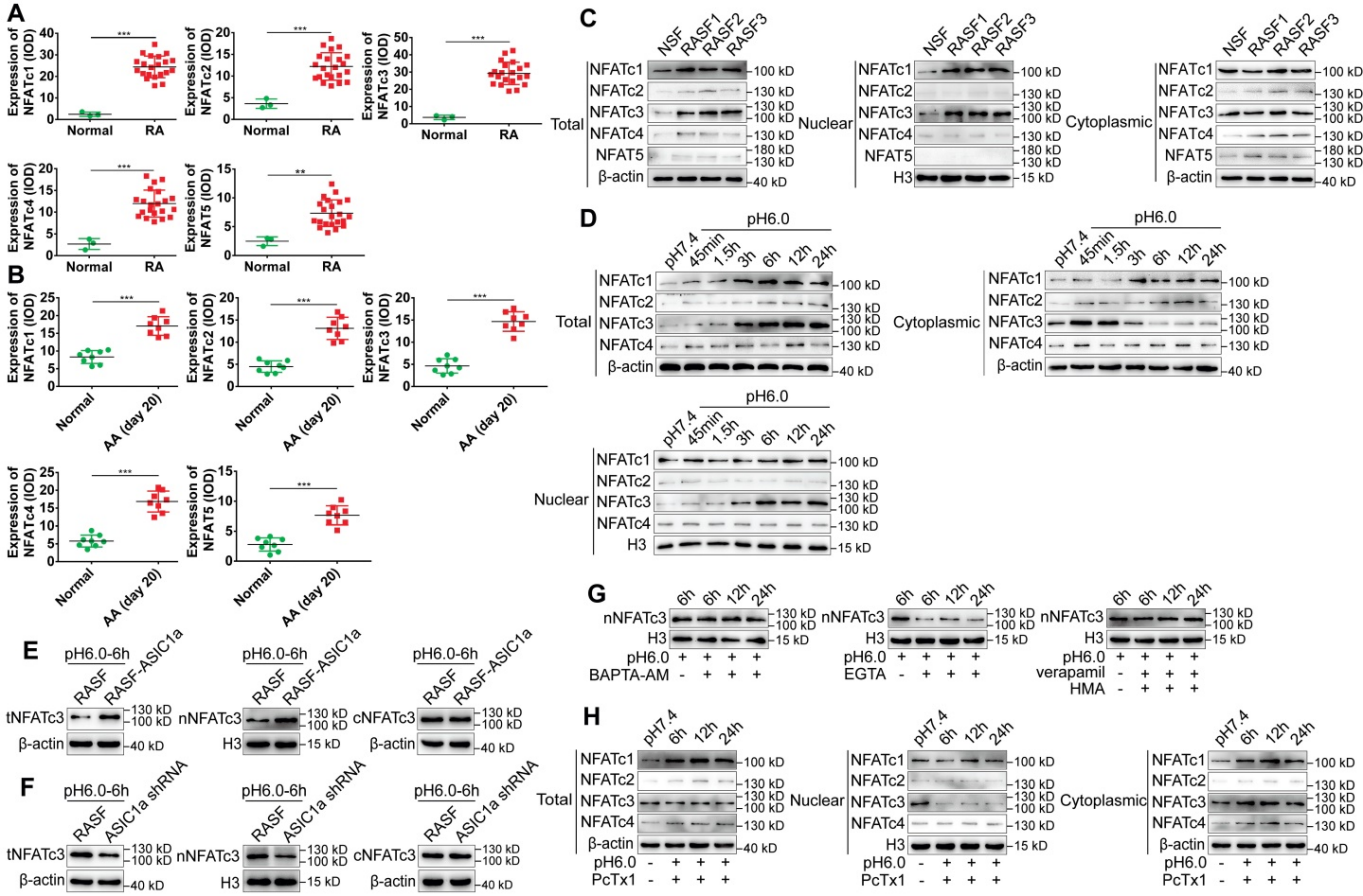

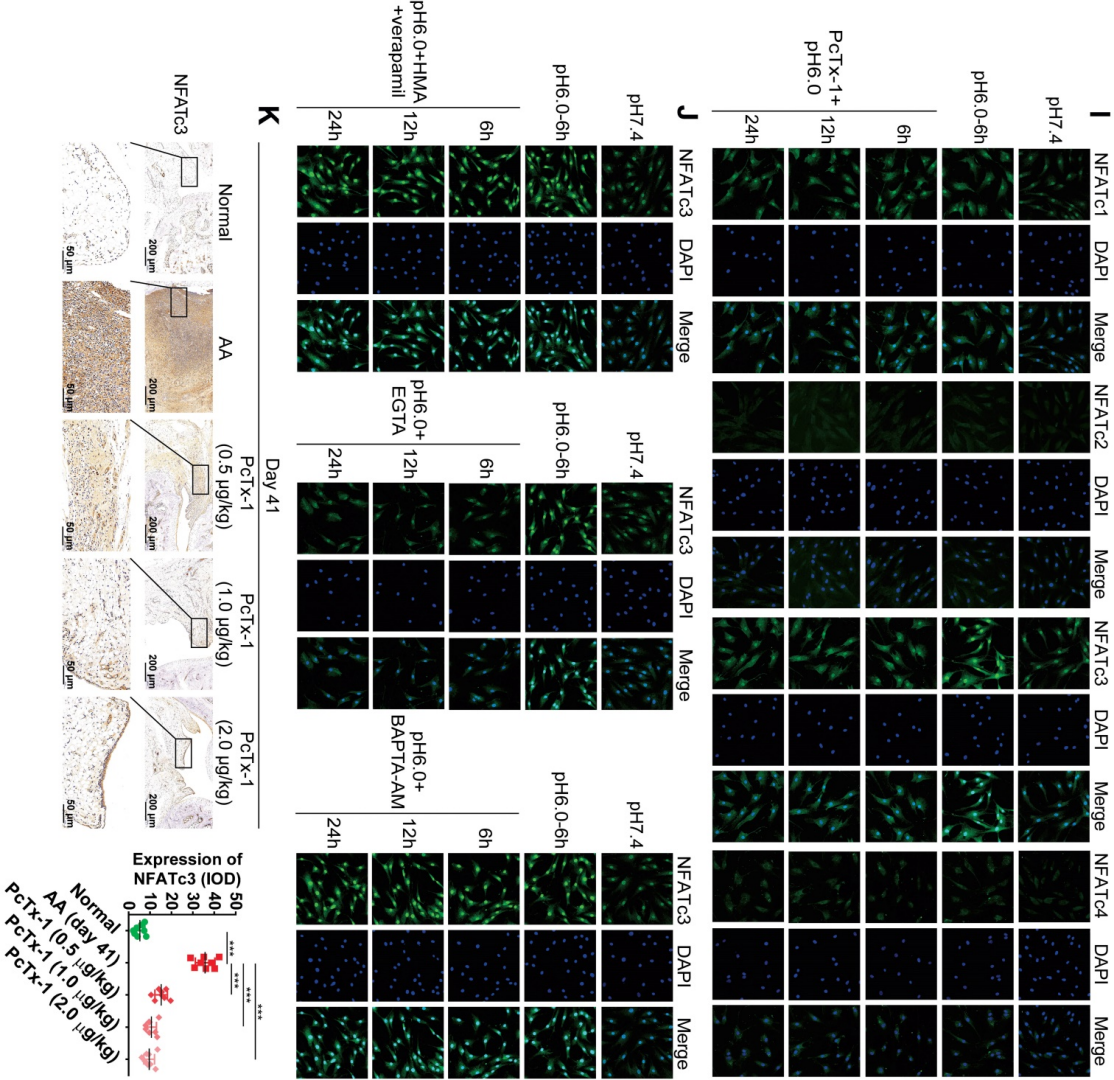
** ASIC1a induces nuclear translocation of NFATc3 by mediating Ca^2+^ influx. (A)** Semi-quantitative analysis of NFATc1-4 and NFAT5 expressions in human normal and RA synovial tissues by integral optical density (IOD).** (B)** Semi-quantitative analysis of NFATc1-4 and NFAT5 expressions in rat ankle synovium by integral optical density (IOD). **(C)** Western blot analysis of total, nuclear, and cytoplasmic NFATc1-4 and NFAT5 expressions in NSF and RASF. **(D)** Western blot analysis of total, nuclear, and cytoplasmic NFATc1-4 expressions in RASF in an acidic environment (pH 6.0). **(E)** Western blot analysis of total NFATc3 (tNFATc3), nuclear NFATc3 (nNFATc3), and cytoplasmic NFATc3 (cNFATc3) expression in RASF-ASIC1a transfectants treated with pH 6.0 for 6 h. **(F)** Western blot analysis of total NFATc3 (tNFATc3), nuclear NFATc3 (nNFATc3) and cytoplasmic NFATc3 (cNFATc3) expression in ASIC1a shRNA transfectants treated with pH 6.0 for 6 h.** (G)** Western blot analysis of Nuclear NFATc3 (nNFATc3) proteins in RASF with 1 mM EGTA, 10 μM BAPTA-AM, 5 μM verapamil and HMA. **(H)** Western blot analysis of total, nuclear and cytoplasmic NFATc1-4 proteins in with 100 nM PcTx-1 were used in the experiment. **(I)** Immunofluorescence analysis of NFATc1-4 expressions in RASF treated with pH 6.0 + 100 nM PcTx-1. The scale bars are 20 μm. **(J)** Immunofluorescence analysis of NFATc3 expression in RASF with 1 mM EGTA, 10 μM BAPTA-AM, 5 μM verapamil and HMA. The scale bars are 20 μm. **(K)** Immunohistochemistry analysis of NFATc3 expression in rat ankle synovium on day 41 (n = 8). Student´s t-test or one-way ANOVA was used for statistical analysis, and data are expressed as mean ± SEM for three separate experiments.

First, the expression levels of NFATc1-4, NFAT5 in human RA synovial tissues and normal synovial tissues were examined by immunohistochemistry and immunofluorescence staining. As displayed in Figures 5A and S4A-B, the expression of NFAT5 was slightly increased but that of NFATc1-4 was significantly increased in RA synovial tissues compared to normal synovial tissues. NFATc1-4, NFAT5 had a co-expression region with CD55, which indicated that NFATc1-4 and NFAT5 were expressed in synovial fibroblasts (Figure S4B). Also, the expression of NFATc1-4 was significantly increased, whereas NFAT5 had a similar but weaker expression in the synovium of AA rats compared with normal rats (Figure 5B and S4C). The expression level of NFATs was detected in RASF and NSF by Western blotting that showed an increase in the expression of total and nuclear NFATc1, c3, while total and nuclear NFATc2, c4 & 5 were slightly increased compared with NSF (Figure 5C and S4F). Immunofluorescence staining showed that nuclear NFATc1 and NFATc3 were remarkably increased and nuclear NFATc2, c4 & 5 were slightly increased in RASF compared with NSF (Figure S4D). These results indicated that the expression of NFATc1 and NFATc3 was significantly increased in tissues and cells and may be related to RA.

Next, we investigated whether ASIC1a could induce the nuclear translocation of NFATc1-4 by mediating Ca^2+^ influx. NFAT5 was not included in the analysis because it was not regulated by Ca^2+^. RASF were cultured in an acidic environment (pH 6.0) for 45 min, 1.5 h, 3 h, 6 h, 12 h, and 24 h. Western blotting and immunofluorescence staining results showed that, compared with the pH 7.4 group, expression of total NFATc1 and c3 gradually increased with time and stabilized after 6 h, whereas total NFATc2 and c4 were slightly increased (Figures 5D, S4D and S4G). However, only the expression of nuclear NFATc3 increased and cytoplasmic NFATc3 decreased with time in the acidic environment, whereas the nuclear proteins of other NFATs were not affected (Figure 5D and S4G). Figure 5H and S4K shows that when ASIC1a activity was inhibited by PcTx-1 in RASF, acid-induced increased level of total NFATc3 expression was significantly attenuated and the expression of nuclear NFATc3 was decreased to a lower level than that of the pH 7.4 group. The expression of total and nuclear NFATc1, c2, & c4 was not affected in RASF treated with PcTx-1 (Figure 5H and S4K). Immunofluorescence staining also showed that the expression of nuclear NFATc3 was remarkably decreased in RASF treated with PcTx-1 compared to the pH 6.0-6 h group, but the nuclear NFATc1, c2, & c4 were not affected (Figure 5I).

Furthermore, compared to RASF, the expression of total and nuclear NFATc3 was considerably increased in RASF-ASIC1a transfectants and decreased in ASIC1a shRNA transfectants by Western blotting (Figure 5E-F and S4H-I) as well as by immunofluorescence staining (Figure S4E). Also, the acid-induced increase of nuclear NFATc3 expression was significantly attenuated by EGTA and was not affected by verapamil and HMA or BAPTA-AM (Figures 5G, 5J, S4E and S4J). Importantly, in the *in vivo* experiment, the expression of NFATc3 was significantly decreased in the synovium of AA rats treated with PcTx-1 compared with AA rats (Figures 5K). These results showed that ASIC1a promoted nuclear translocation of NFATc3 by enhancing Ca^2+^ influx.

### ASIC1a induces nuclear translocation of NFATc3 to regulate transcription of the* RANTES* gene

RANTES, also known as chemokine C-C motif ligand 5 (CCL5), which is classified as a chemokine encoded by the *CCL5* gene, and acts as an important inflammatory cytokine that promotes the progression of RA 51-54. Our results have shown that acid-induced activation of ASIC1a enhanced RANTES expression by mediating Ca^2+^ influx, and its effect was significantly stronger than that of IL-8, MIP-1a, ICAM-1, TNF RI, and TNF RII. Moreover, ASIC1a induced nuclear translocation of NFATc3 by mediating Ca^2+^ influx. Therefore, it was essential to investigate whether ASIC1a induced the nuclear translocation of NFATc3 to regulate transcription of the* RANTES* gene.

**Figure 6:**
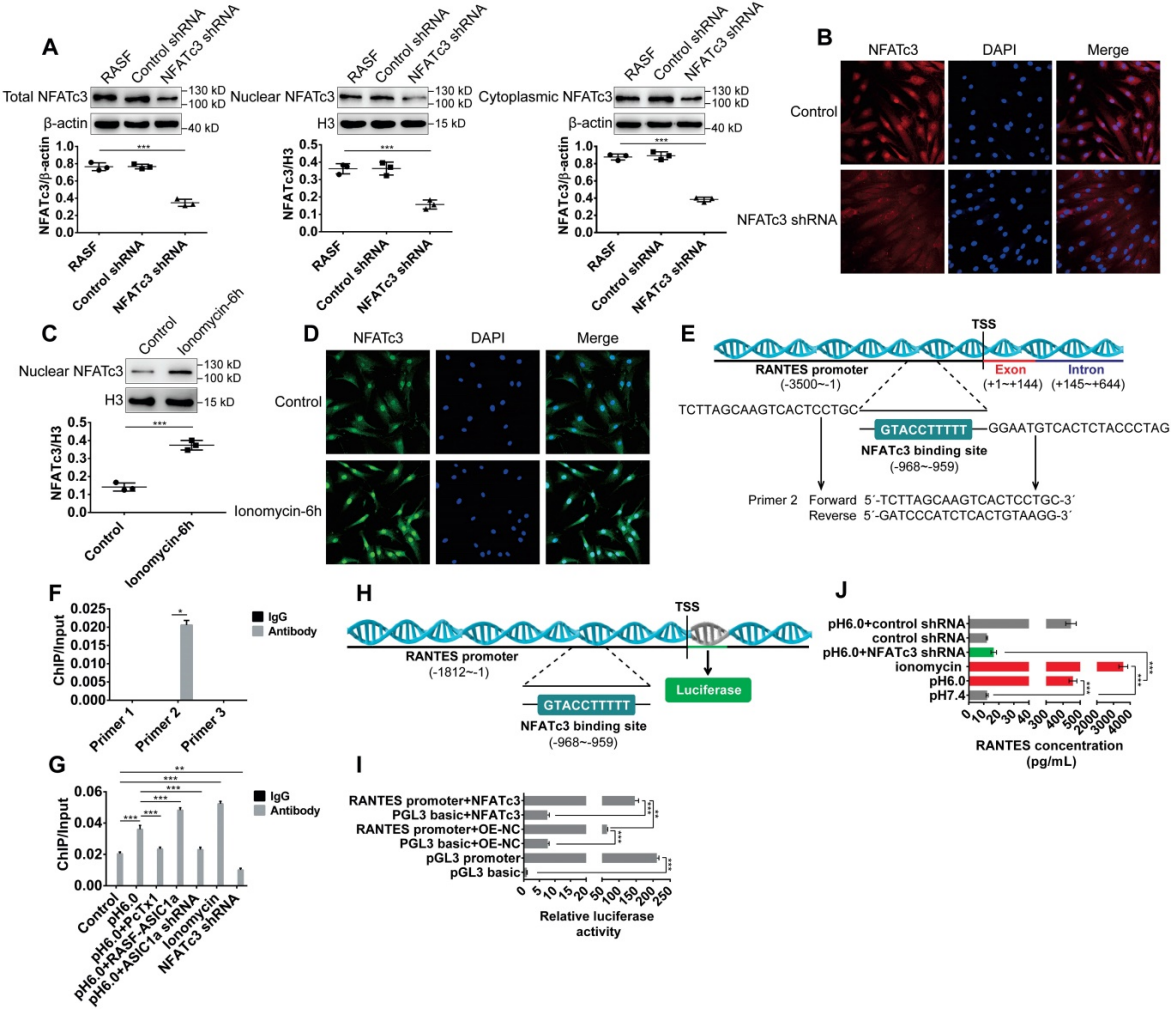
** ASIC1a induces nuclear translocation of NFATc3 to regulate transcription of the RANTES gene. (A)** Western blot analysis of total, nuclear, and cytoplasmic NFATc3 in NFATc3 shRNA transfectants. **(B)** Immunofluorescence analysis of NFATc3 expression in NFATc3 shRNA transfectants. The scale bars are 20 μm. **(C)** Western blot analysis of nuclear NFATc3 in RASF treated with 5μM ionomycin. **(D)** Immunofluorescence analysis of NFATc3 expression in RASF treated with 5μM ionomycin. The scale bars are 20 μm. **(E)** Schematic diagram of the primer 2-guided amplified DNA fragments (1005, 902) and predicted binding site for NFATc3 in the promoter region of the human *RANTES* gene. Transcription start site (TSS) of the RANTES promoter is +1. **(F)** Primer 1, 2, and 3 were used to detect the binding of NFATc3 to RANTES promoter region in untreated RASF by ChIP-qPCR assay. **(G)** Primer 2 was used to detect the binding of NFATc3 to RANTES promoter region in treated RASF by ChIP-qPCR assay in the presence of 5 μM ionomycin. **(H)** Schematic diagram of the promoter region of the human *RANTES* gene used for dual-luciferase reporter assay. Transcription start site (TSS) of the RANTES promoter is +1. **(I)** Dual-luciferase reporter assay showing regulation of RANTES transcription by direct binding of NFATc3 to the RANTES promoter region in RASF. pGL3 basic group and pGL3 promoter group served as negative and positive controls, respectively. **(J)** The expression of RANTES was determined in RASF treated with 5 μM ionomycin and NFATc3 shRNA transfectants by magnetic multi-cytokine assay. Student´s t-test or one-way ANOVA was used for statistical analysis, and data are expressed as mean ± SEM for three separate experiments.

We first silenced NFATc3 by using shRNA-lentiviruses. Western blots and immunofluorescence analysis showed that the nuclear translocation of NFATc3 protein was significantly decreased in NFATc3 shRNA transfectants compared to the control transfectants (Figure 6A-B). NFATc3 was activated by ionomycin that is a membrane-permeable Ca^2+^ ionophore. As shown in Figure 6C-D, nuclear translocation of NFATc3 was increased in RASF treated with 5 μM ionomycin. The NCBI gene database was used to search the human RANTES genomic sequence, and the human RANTES promoter sequence was predicted using the UCSC genome bioinformatics program (Table S3). As reported previously, the NFATc3 binding motif consisted of TTTCC or GGAAA 55. We designed three pairs of primers to detect the promoter regions of RANTES (Table S3) and investigated the binding of NFATc3 to the RANTES promoter region by ChIP-qPCR assay. The results showed that primer pair 2 could direct the amplification of DNA obtained by ChIP in untreated RASF, while primer pairs 1, 3 could not (Figure 6F).

The binding sites for NFATc3 in the RANTES gene promoter were predicted using the JASPAR database (http://jaspar.genereg.net, Table S5), which revealed 22 putative binding sites and provided a quantitative score for each binding site. The original score was normalized to a range of 0-1 to provide a relative score. The relative score of one putative binding site was 0.963, which was the highest compared to other binding sites. The sequence of this binding site was TTTTTCCATG, which was located at -968 bp in the promoter, relative to the -959 bp transcription start site, and was also located in primer pair 2-guided amplified DNA fragments (-1005 ~ -902, Figure 6E). Compared with the control group, NFATc3 binding to the RANTES promoter was markedly up-regulated by acid-induced activation of ASIC1a but was considerably reduced by silencing of ASIC1a or administration of PcTx-1 (Figure 6G). Similarly, compared with the control, it was significantly increased by administration of ionomycin and was markedly decreased by silencing of NFATc3 in RASF (Figure 6G). These data indicated that NFATc3 bound to the RANTES promoter region and was regulated by ASIC1a.

Next, we investigated whether NFATc3 directly regulated RANTES transcription by binding to the RANTES promoter region by dual-luciferase reporter assay. We amplified 1812 bp relative to the transcriptional start site gene region upstream of the RANTES promoter by PCR to generate RANTES promoter constructs (Figure 6H). As shown in Figure 6I, compared with the control plasmid transfection pGL3 basic+OE-NC group (pGL3 basic is a control plasmid of RANTES promoter plasmid and OE-NC is a control plasmid of NFATc3 overexpression plasmid), RANTES promoter activity was significantly increased by transfection of RANTES promoter plasmid (RANTES promoter+OE-NC group) in RASF. Furthermore, compared with RANTES promoter+OE-NC group and pGL3 basic+NFATc3 group, RANTES promoter activity was significantly increased by co-transfection of RANTES promoter plasmid and NFATc3 overexpression plasmid (RANTES promoter+NFATc3 group) in RASF indicating that NFATc3 directly regulated RANTES transcription (Figure 6I). The magnetic multi-cytokine assay showed that acid-induced increase of RANTES expression was significantly attenuated by silencing of NFATc3 compared with pH 6.0 group, and RANTES expression was increased by administration of ionomycin compared with pH 7.4 group (Figure 6J). Thus, ASIC1a mediated nuclear translocation of NFATc3 that bound to the RANTES promoter and directly regulated RANTES transcription.

## Discussion

RA is one of the most common chronic inflammatory diseases that cause disability due to uncontrolled inflammation 56-60. The etiology of rheumatoid arthritis has not yet been elucidated due to the crosstalk between multiple factors such as susceptibility genes, epigenetic modifications, environmental insults, and post-translational modifications. The lack of molecular understanding of the disease leads to the current untargeted treatment strategy that only relieves symptoms 7, 61, 62. Early RA manifests itself as synovial inflammation that is characterized by infiltration of inflammatory cells such as monocytes, lymphocytes, and neutrophils 63, 64. Synovial inflammation is seen as a robust tissue response that drives RA progression by causing articular cartilage and bone destruction 65, 66. Therefore, early treatment of synovial inflammation might be highly effective in preventing the manifestation of RA 61. However, due to the lack of mechanistic understanding, there is no effective treatment to control synovial inflammation. Previous studies on synovial inflammation have focused on immune cells rather than RASF 63, 64, 67. As one of the main constituent cells in RA synovial tissues, RASF assume an aggressive inflammatory, matrix regulatory, and invasive phenotype, which, together with enhanced chondrocyte catabolism and synovial osteoclastogenesis, promotes articular destruction 66, 68. This suggests that RASF are a critical component of the synovial tissue to investigate the mechanism of synovial inflammation.

In mammals, the pH values of blood and tissues are usually maintained in a narrow range around 7. 4, which is primarily regulated by respiration and renal acid excretion 69, 70. When intracellular contents leak or blood vessels are damaged, extracellular pH drops to a value below 6, which results in hypoxic metabolism and related lactic acid production during inflammation to resist pathogen infection 71. Mounting evidence has revealed that extracellular acidification mediates inflammation by modulating inflammatory cells such as monocytes, macrophages, and dendritic cells 72-74 and implies that extracellular acidification might be involved in synovial inflammation. On the other hand, our previous studies have found that extracellular acidification induces cartilage destruction by activating ASIC1a, which leads to the progression of RA 28-30. As an acid receptor on the cell membrane, ASIC1a transmits the signal of the low pH of the extracellular microenvironment into the cell to activate downstream signaling pathways and produces a series of physiological and pathological changes. Many studies have observed that a low extracellular pH triggers endocytosis and maturation of macrophages and dendritic cells by activating ASICs, suggesting a possible role for ASICs in the inflammation process 31, 75. Based on these observations, we hypothesized that extracellular acidification induces synovial inflammation through ASIC1a that acts as a membrane receptor. Thus, we investigated the function and molecular mechanism of ASIC1a in synovial inflammation.

We found markedly increased expression level of ASIC1a in human RA synovial tissues compared with normal synovial tissue and in AA rats compared with normal rats, which suggested that ASIC1a may be involved in the pathological process of RA. Similarly, the expression of ASIC1a was significantly upregulated in RASF compared with NSF. Acid-induced activation of ASIC1a markedly enhanced RANTES expression by mediating Ca^2+^ influx in RASF. ASIC1a also promoted the expressions of IL-8, MIP-1a, ICAM-1, sTNF RI, and sTNF RII, but this effect was remarkably weaker than that of RANTES. Importantly, inhibition of ASIC1a could control the development of joint inflammation *in vivo* and prevented cartilage and bone destruction. To sum up, ASIC1a is a vital mediator of synovial inflammation.

NFATs are a group of Ca^2+^-dependent transcription factors, which regulate the transcription of inflammation-related genes to drive the inflammatory process. Many studies have found that NFATs mediate the expression of many inflammatory cytokines such as IL-2, IL-17, GM-CSF, IL-4, and IL-5 during inflammation 40-44, 76. Other studies reported that NFATc1 promotes osteoclastogenesis to mediate bone loss, thereby promoting the progression of RA 77, 78. It is of note that NFAT5 promotes macrophage survival to enhance chronic inflammation in RA 45. These studies have shown that NFATs play a facilitator role in RA.

Other studies, for example, by Laura V et al. reported that ASIC1a induces nuclear translocation of NFATc3 by mediating Ca^2+^ influx in pulmonary arterial smooth muscle cells, and Li et al. have found that ASIC1a is involved in acid-induced osteoclastogenesis by regulating activation of NFATc1 79, 80. Therefore, we speculated that ASIC1a induces nuclear translocation of NFATs that drives the inflammatory process by regulating the transcription of inflammatory cytokine *RANTES* gene in synovial inflammation. Indeed, we observed significantly higher expression levels of the NFATc1-4 in RA synovial tissues compared to normal synovial tissues, whereas NFAT5 expression was slightly elevated. Similar expression level changes of NFATs were also observed in RASF. Further, we found that ASIC1a mediated nuclear translocation of NFATc3 that bound to the RANTES promoter to directly regulate *RANTES* gene transcription. In summary, ASIC1a drives synovial inflammation by inducing NFATc3-regulated RANTES transcription.

Chemokines are a class of central cytokine proteins, which are involved in inflammatory responses and induce leukocyte recruitment, activate integrins, stimulate mediator release, and modulate vascularization 81, 82. In RA, chemokines induce recruitment and retention of neutrophils, lymphocytes, and monocytes in the joints, leading to inflammation and hyperplasia of the synovium and causing destruction of bone and articular cartilage 83-85. As an important chemokine, RANTES induces the recruitment of many inflammatory cells such as dendritic cells, monocytes, mast cells, neutrophils, macrophages, and lymphocytes. RANTES has been associated with a wide range of inflammatory disorders and pathologies, including RA, allogeneic transplant rejection, atherosclerosis, atopic dermatitis, inflammatory airway disorders such as asthma, delayed-type hypersensitivity reactions, glomerulonephritis, endometriosis, some neurological disorders, and certain malignancies 86, 87. Some studies have found that RANTES was increased in synovial tissues and synovial fluids 88, 89. Furthermore, RANTES induced Th1 cells and leukocytes recruitment to trigger synovial inflammation and induces collagen degradation by activating the expression of MMP-1 and MMP-13 52, 90-92.

**Figure 7:**
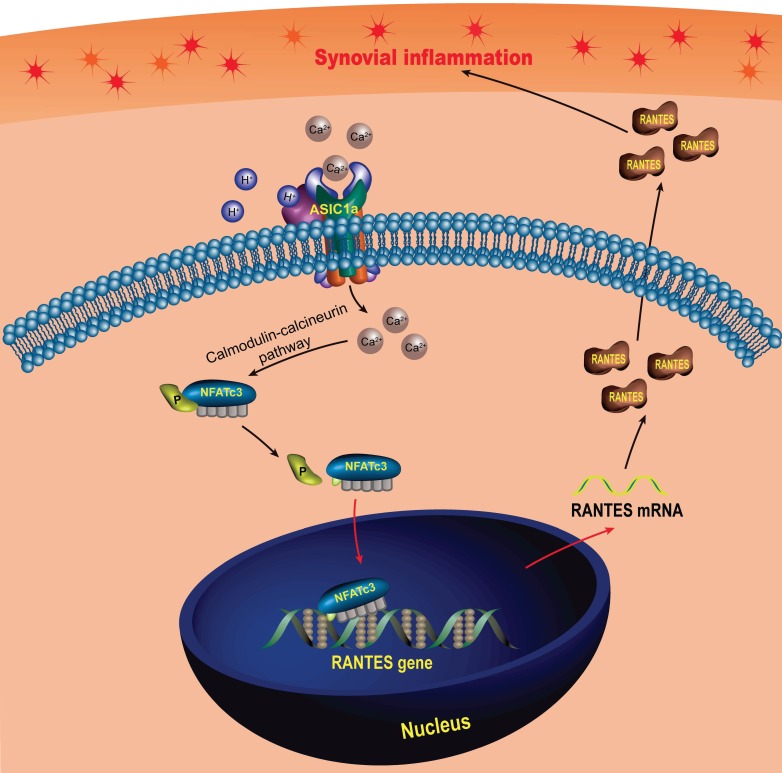
** Schematic diagram of the molecular mechanism of ASIC1a-induced synovial inflammation.** ASIC1a mediates Ca^2+^ influx to enhance nuclear translocation of NFATc3, which binds to the RANTES promoter to directly regulate RANTES gene transcription and protein expression.

In this study, we found that RANTES was produced by RASF, which was consistent with previous findings 93, 94. Above all, we not only confirmed the role of RANTES as an inflammation inducer to promote the progression of RA, but also elucidated the mechanisms regulating its expression. Our research showed that extracellular acidification activated ASIC1a to mediate nuclear translocation of NFATc3 that bound to the RANTES promoter to directly regulate RANTES transcription and enhance its expression, which was involved in the induction of synovial inflammation (Figure 7). These findings imply that ASIC1a could be a potential therapeutic target for preventing synovial inflammation and controlling the progression of RA. Furthermore, our study revealed extracellular acidification as a crucial pathogenic determinant of synovial inflammation providing new insight into the pathogenesis of RA. However, ASIC1a acts as an ion channel on the cell membrane and may have a broader role in cellular physiology and pathology. Therefore, whether ASIC1a regulates other potential mechanisms in synovial inflammation remains to be further studied.

## Supplementary Material

Supplementary figures and tables.

## Abbreviations

AA: adjuvant arthritis
ANOVA: analysis of variance
ASICs: Acid sensing ion channels
ASIC1a: acid sensing ion channel 1a
BAPTA-AM: 1,2-Bis(2-aminophenoxy)ethane-N,N,N′,N′-tetraacetic acid tetrakis
[Ca^2+^]i: intracellular free calcium concentration
CCL5: chemokine C-C motif ligand 5
CFA: Complete Freund's adjuvant
DAPI: 4′,6-Diamidino-2-phenylindole dihydrochloride
EGTA: ethylene glycol tetraacetic acid
Fluo 3-AM: Fluo 3 acetoxymethyl ester
GM-CSF: granulocyte macrophage colony stimulating factor
HMA: 5-(N,N-Hexamethylene) amiloride
ICAM-1: intercellular cell adhesion molecule-1
IL-8: interleukin 8
MFI: mean fluorescence intensity
MIP-1a: Macrophage inflammatory protein-1 alpha
NFATs: nuclear factor of activated T cells
NSF: normal synovial fibroblasts
PCR: polymerase chain reaction
PcTx-1: psalmotoxin 1
RA: rheumatoid arthritis
RANTES: Regulated upon activation, normal T cell expressed and secreted
RASF: rheumatoid arthritis synovial fibroblasts
SEM: standard error of mean
shRNA: short hairpin RNA
sTNF RI: soluble tumor necrosis factor receptor 1
sTNF RII: soluble tumor necrosis factor receptor 2
