# Supplementary material for: ASIC1a induces synovial inflammation via the Ca2+/NFATc3/ RANTES pathway
Source: Theranostics. 2020 Jan 1;10(1):247–64. doi: 10.7150/thno.37200 (PMC6929608; doi:10.7150/thno.37200)
Supplement: Supplementary file 1 — Supplementary figures and tables. [file thnov10p0247s1.pdf]

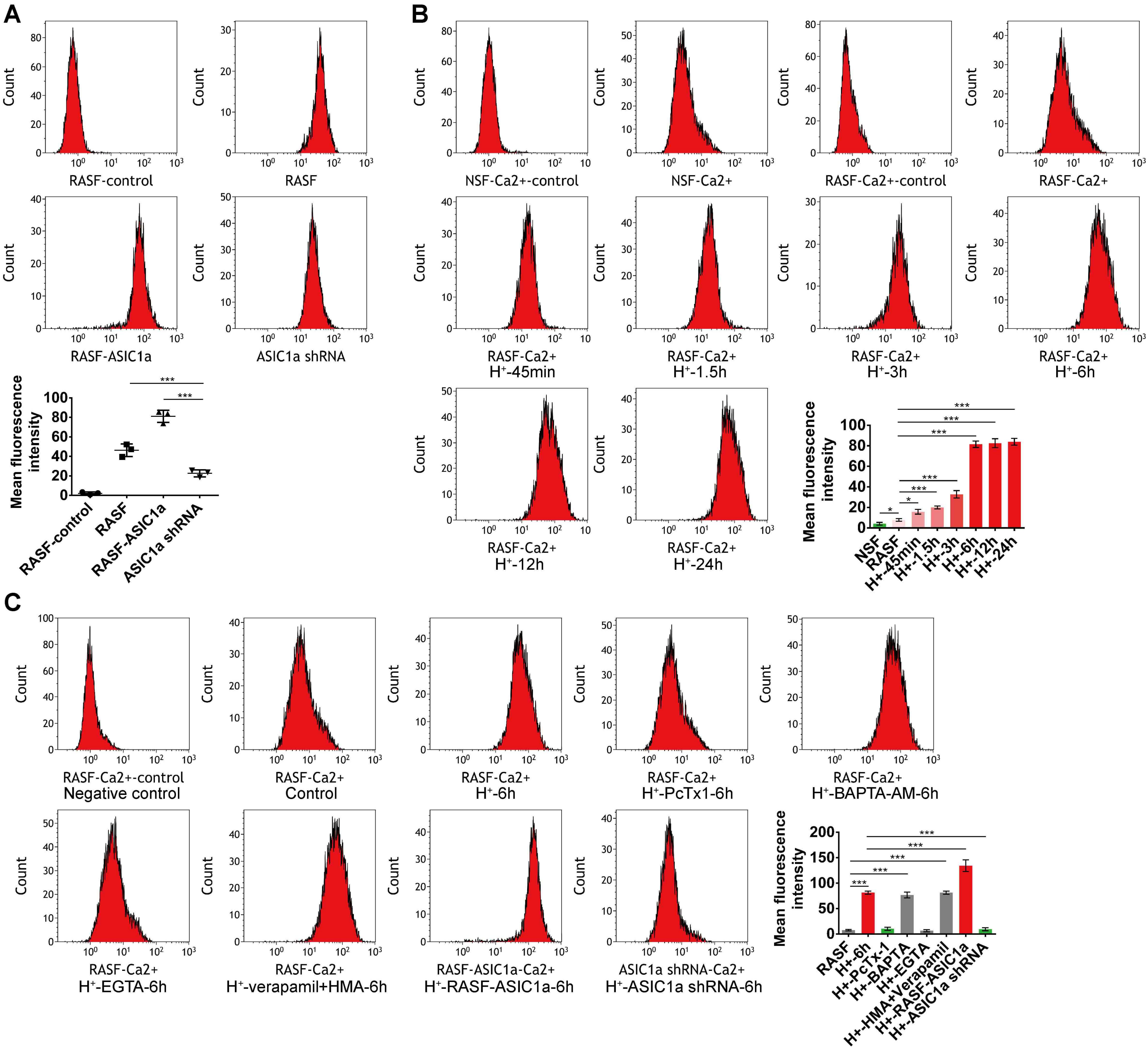

Figure S1. ASIC1a has an ion channel activity that mediates Ca^{2+} influx. (A) Membrane ASIC1a expression was detected in RASF-ASIC1a transfectants and ASIC1a shRNA transfectants by flow cytometry. **(B)** The effect of ASIC1a on $[Ca^{2+}]$i was detected in RASF with acidic solution (pH 6.0) by flow cytometry . **(C)** Flow cytometry detected that the increase of $[Ca^{2+}]$i was mediated by ASIC1a in RASF rather than by intracellular Ca^{2+} stores release or other calcium channels. 100 nM PcTx-1, 1 mM EGTA, 10 μM BAPTA-AM, 5 μM verapamil and HMA were used in the experiment. Student´s t-test or one-way ANOVA was used for statistical analysis and data are expressed as mean ± SEM for three separate experiments.

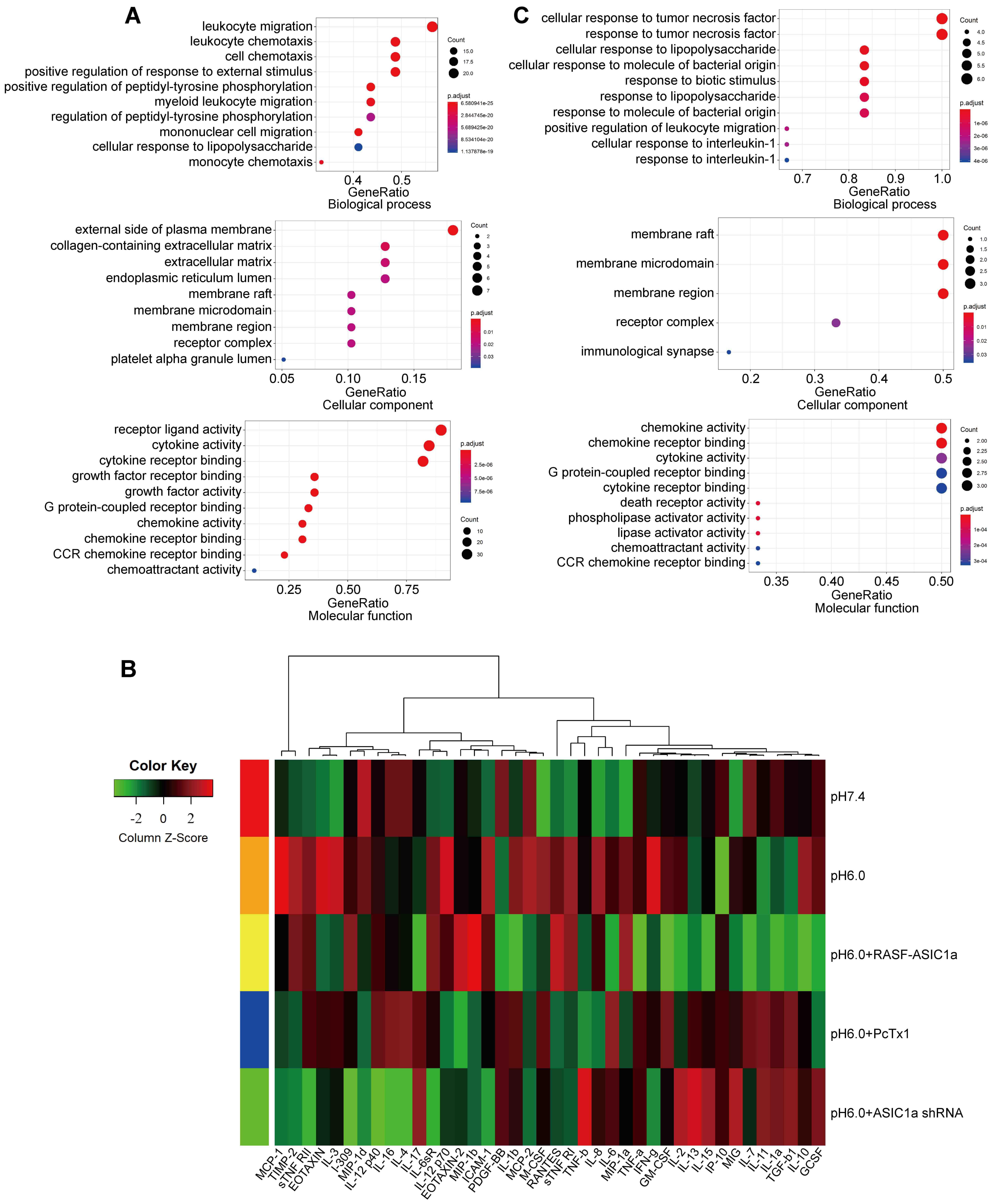

A
leukocyte migration
leukocyte chemotaxis
cell chemotaxis
positive regulation of response to external stimulus
positive regulation of peptidyl-tyrosine phosphorylation
myeloid leukocyte migration
regulation of peptidyl-tyrosine phosphorylation
mononuclear cell migration
cellular response to lipopolysaccharide
monocyte chemotaxis
GeneRatio
Biological process
external side of plasma membrane
collagen-containing extracellular matrix
extracellular matrix
endoplasmic reticulum lumen
membrane raft
membrane microdomain
membrane region
receptor complex
platelet alpha granule lumen
GeneRatio
Cellular component
receptor ligand activity
cytokine activity
cytokine receptor binding
growth factor receptor binding
growth factor activity
G protein-coupled receptor binding
chemokine activity
chemokine receptor binding
CCR chemokine receptor binding
chemoattractant activity
GeneRatio
Molecular function
C
cellular response to tumor necrosis factor
response to tumor necrosis factor
cellular response to lipopolysaccharide
cellular response to molecule of bacterial origin
response to biotic stimulus
response to lipopolysaccharide
response to molecule of bacterial origin
positive regulation of leukocyte migration
cellular response to interleukin-1
response to interleukin-1
GeneRatio
Biological process
membrane raft
membrane microdomain
membrane region
receptor complex
immunological synapse
GeneRatio
Cellular component
chemokine activity
chemokine receptor binding
cytokine activity
G protein-coupled receptor binding
cytokine receptor binding
death receptor activity
phospholipase activator activity
lipase activator activity
chemoattractant activity
CCR chemokine receptor binding
GeneRatio
Molecular function
Count
p.adjust
B
Color Key
Column Z-Score
pH7.4
pH6.0
pH6.0+RASF-ASIC1a
pH6.0+PcTx1
pH6.0+ASIC1a shRNA
MCP-1
TIMP-2
sTNF RII
EOTAXIN
IL-3
I-309
MIP-1d
IL-12 p40
IL-16
IL-4
IL-17
IL-6sR
IL-12 p70
EOTAXIN-2
MIP-1b
ICAM-1
PDGF-BB
IL-1b
MCP-2
M-CSF
RANTES
sTNF RI
TNF-b
IL-8
IL-6
MIP-1a
TNF-a
IFN-g
GM-CSF
IL-2
IL-13
IL-15
IP-10
MIG
IL-7
IL-11
IL-1a
TGF-b1
IL-10
GCSF

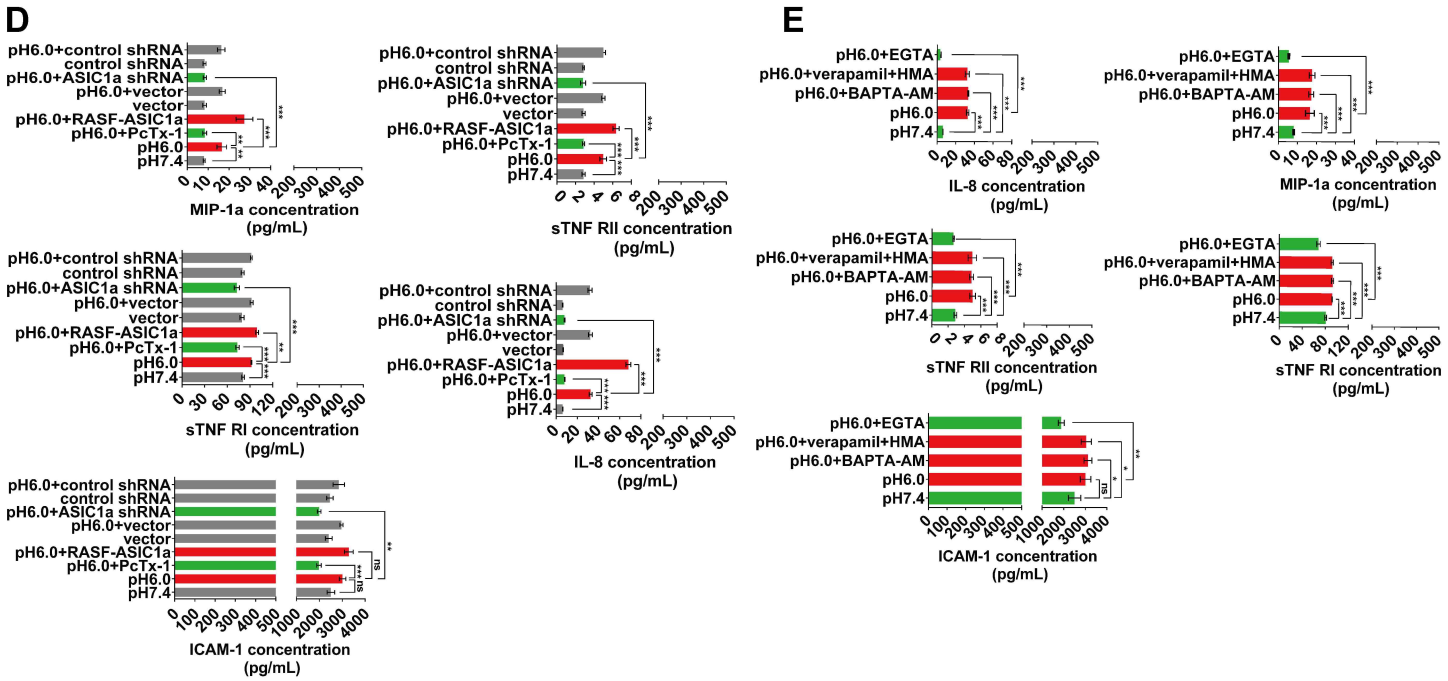

Figure S2. ASIC1a upregulates the expression of inflammatory cytokines by mediating Ca^{2+} influx. (A) Molecular function, biological process and cellular component of 40 inflammatory cytokines were analyzed by Gene Ontology (GO) enrichment analysis. **(B)** The expression of 40 inflammatory cytokines were semi-quantitative analysis by fluorescence intensity and were presented by heat map. **(C)** Molecular function, biological process and cellular component of RANTES, sTNF RI, MIP-1a, IL-8, sTNF RII and ICAM-1 were analyzed by GO enrichment analysis. Student´s t-test or one-way ANOVA was used for statistical analysis and data are expressed as mean ± SEM for three separate experiments. **(D)** The expressions of sTNF RI, IL-8, sTNF RII, and ICAM-1 were determined by magnetic multi-cytokine assay, and the expression of MIP-1a was determined by ELISA. Cells were treated for 6 h with100 nM PcTx-1. **(E)** Magnetic multi-cytokine assay or ELISA detected the increase of sTNF RI, IL-8, sTNF RII, ICAM-1, and MIP-1a expressions mediated by ASIC1a in RASF treated for 6 h with 1 mM EGTA, 10 µM BAPTA-AM, 5 µM verapamil and HMA. Student´s t-test or one-way ANOVA was used for statistical analysis, and data are expressed as mean ± SEM for three separate experiments.

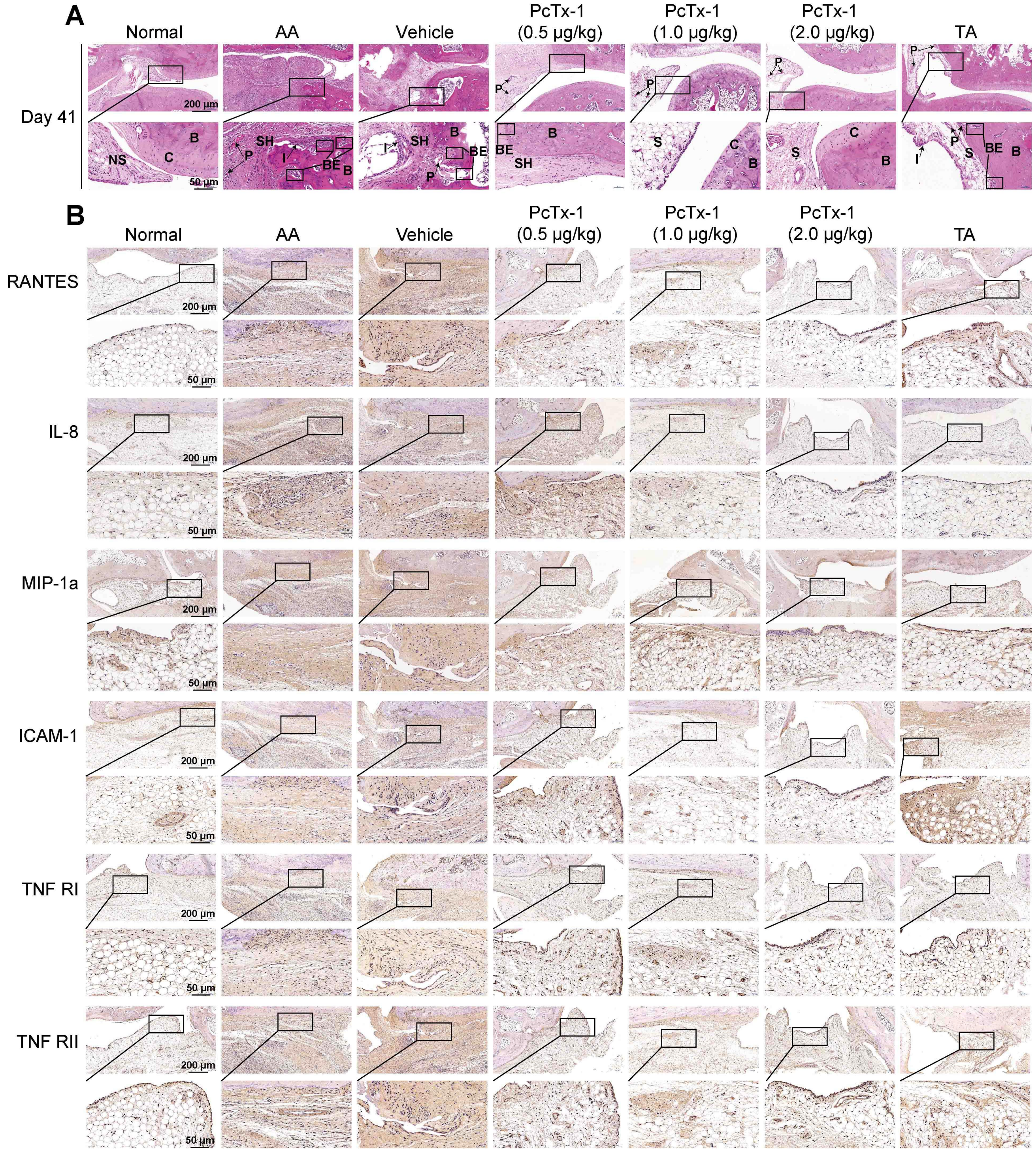

A
Normal
AA
Vehicle
PcTx-1 (0.5 μg/kg)
PcTx-1 (1.0 μg/kg)
PcTx-1 (2.0 μg/kg)
TA
Day 41
200 μm
50 μm
NS
C
B
P
SH
I
BE
S
B
Normal
AA
Vehicle
PcTx-1 (0.5 μg/kg)
PcTx-1 (1.0 μg/kg)
PcTx-1 (2.0 μg/kg)
TA
RANTES
IL-8
MIP-1a
ICAM-1
TNF RI
TNF RII
200 μm
50 μm

C

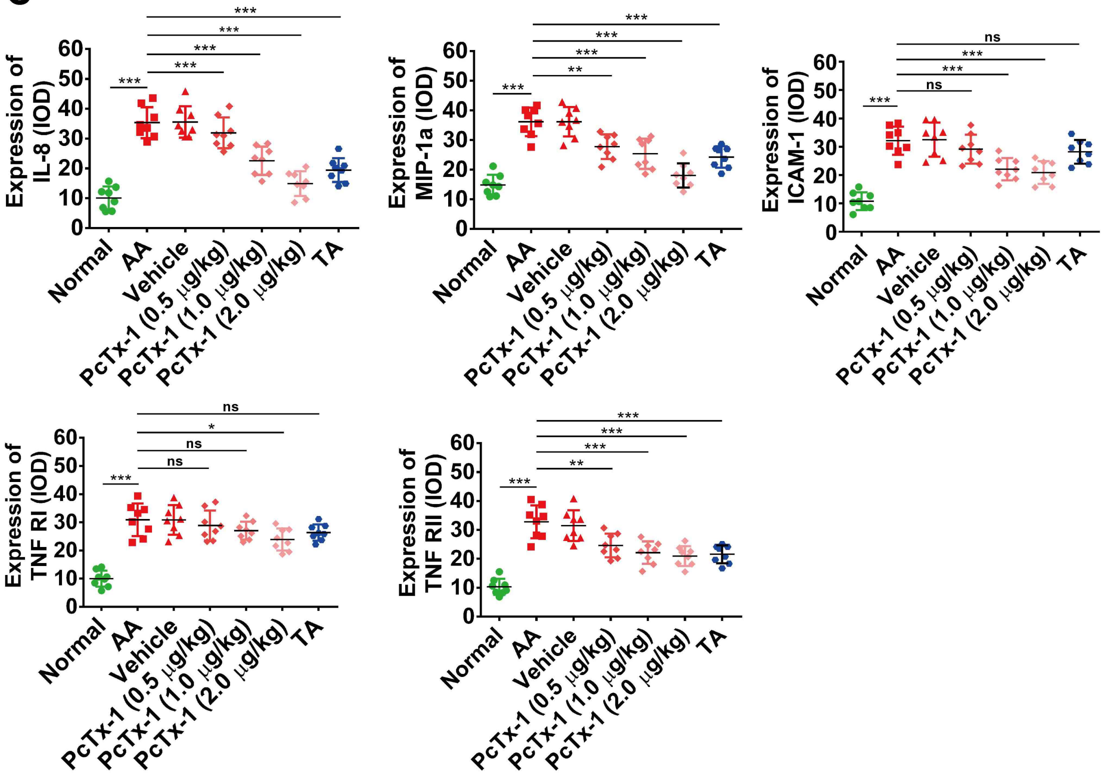

Figure S3. ASIC1a promotes inflammation by mediating the expression of inflammatory cytokines in vivo. **(A)** Experimental rat right hind ankle joint sections stained with hematoxylin and eosin. Histological section showing subchondral bone (B), bone erosion (BE), articular cartilage (C), inflammatory cell infiltration (I), synovium (S), normal synovium (NS), pannus formation (P), synovial hyperplasia (SH). **(B)** Immunohistochemistry analysis of RANTES, TNF RI, MIP-1a, IL-8, TNF RII and ICAM-1 expression in rat ankle synovium (n = 8). **(C)** Semi-quantitative analysis of TNF RI, MIP-1a, IL-8, TNF RII, and ICAM-1 expression in rat ankle synovium by integral optical density (IOD). Student´s t-test or one-way ANOVA was used for statistical analysis, and data are expressed as mean ± SEM for three separate experiments.

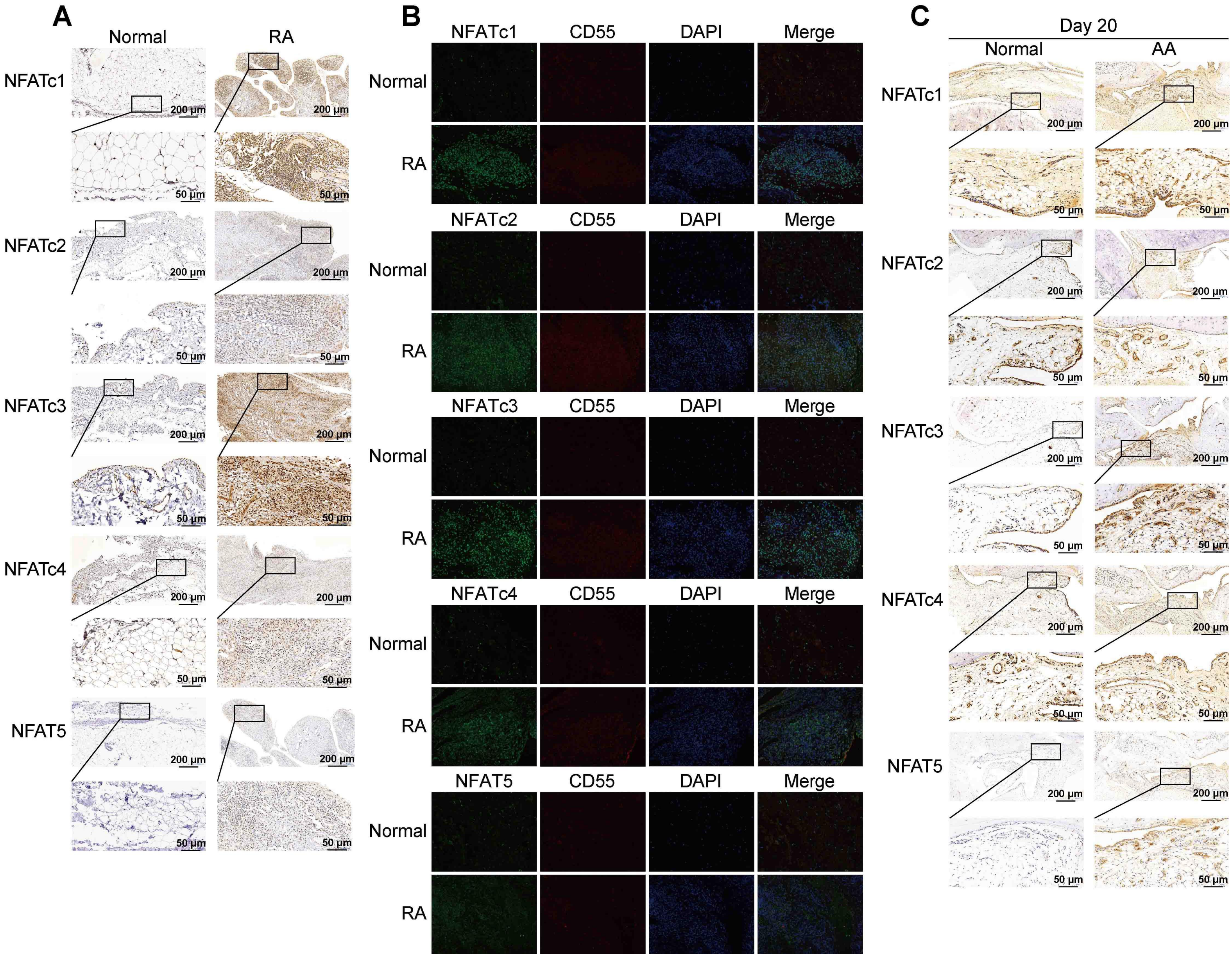
A
Normal
RA
NFATc1
NFATc2
NFATc3
NFATc4
NFAT5
200 μm
50 μm
B
NFATc1
NFATc2
NFATc3
NFATc4
NFAT5
CD55
DAPI
Merge
Normal
RA
C
Day 20
Normal
AA

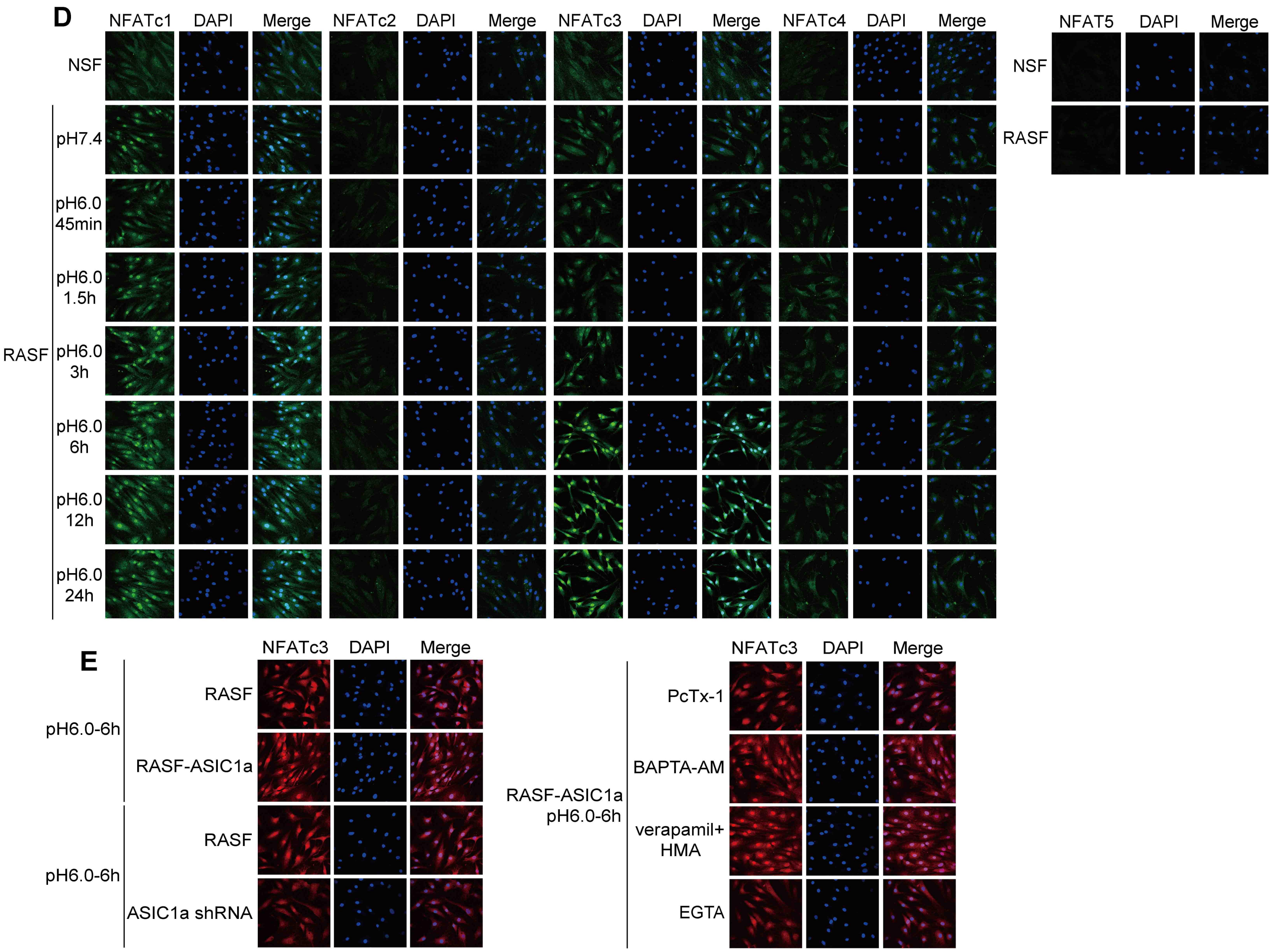
D
NFATc1
DAPI
Merge
NFATc2
DAPI
Merge
NFATc3
DAPI
Merge
NFATc4
DAPI
Merge
NSF
RASF
pH7.4
pH6.0 45min
pH6.0 1.5h
pH6.0 3h
pH6.0 6h
pH6.0 12h
pH6.0 24h
NFAT5
DAPI
Merge
NSF
RASF
E
NFATc3
DAPI
Merge
pH6.0-6h
RASF
RASF-ASIC1a
pH6.0-6h
RASF
ASIC1a shRNA
NFATc3
DAPI
Merge
RASF-ASIC1a pH6.0-6h
PcTx-1
BAPTA-AM
verapamil+ HMA
EGTA

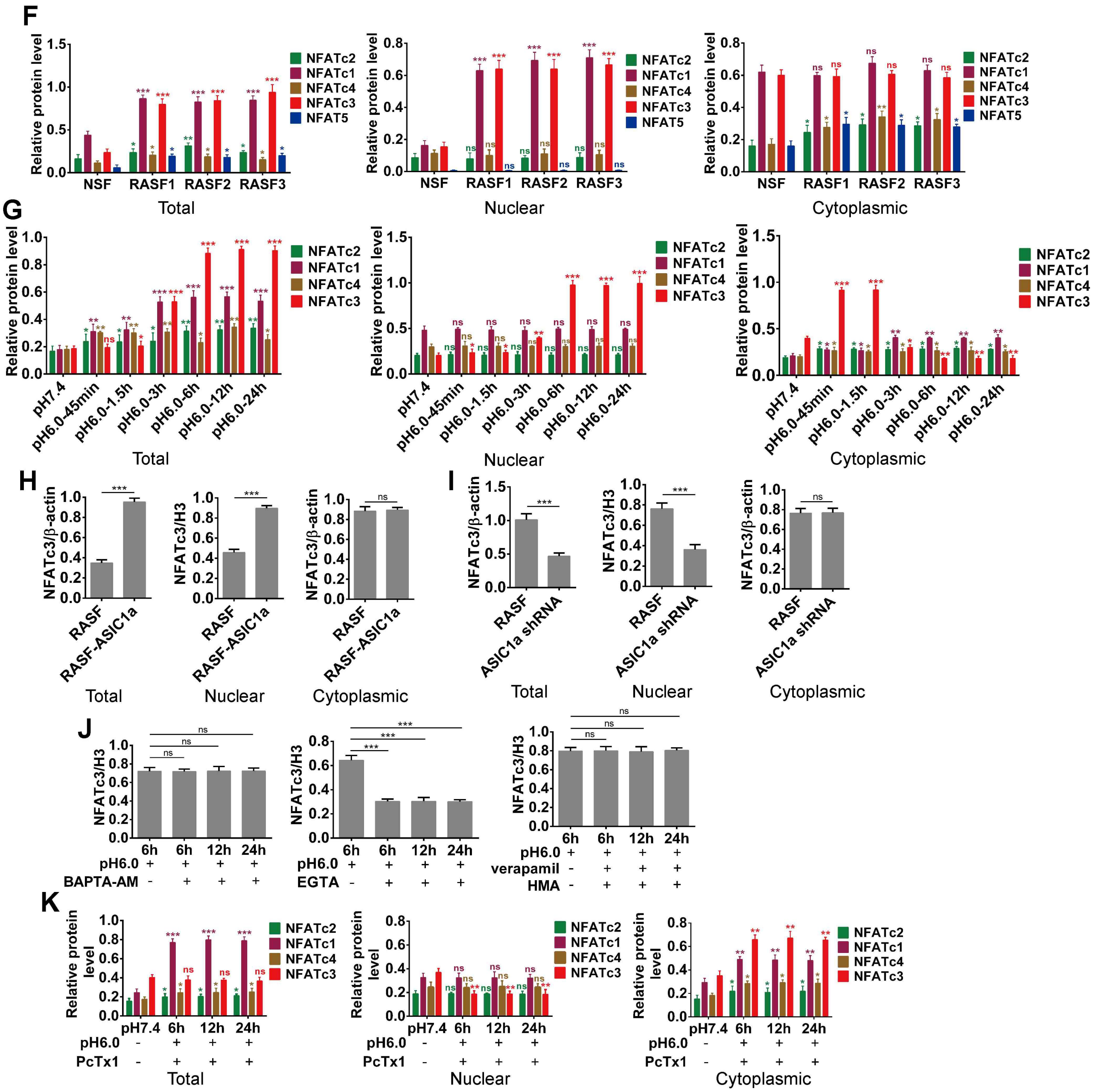

Figure S4. ASIC1a induces the nuclear translocation of NFATc3 by mediating Ca^{2+} influx. (A) Immunohistochemistry analysis of NFATc1-4, NFAT5 expressions in human normal and RA synovial tissues. **(B)** Immunofluorescence analysis of NFATc1-4, NFAT5 expressions in human normal and RA synovial tissues. The scale bars are 100 μm. **(C)** Immunohistochemistry analysis of NFATc1-4, NFAT5 expressions in rat ankle synovium on day 20 (n = 8). **(D)** Immunofluorescence analysis of NFATc1-4, NFAT5 expressions in NSF and RASF. The scale bars are 20 μm. **(E)** Immunofluorescence analysis of NFATc3 expression in RASF-ASIC1a transfectants and ASIC1a shRNA transfectants. The scale bars are 20 μm. **(F)** Semi-quantitative analysis of total, nuclear and cytoplasmic NFATs bands from Western blotting by gray value in NSF and RASF. **(G)** Semi-quantitative analysis of total, nuclear and cytoplasmic NFATc1-4 bands from Western blotting by gray value. RASF treated with pH 6.0 for 45 min, 1.5 h, 3 h, 6 h, 12 h, 24 h. **(H)** Semi-quantitative analysis of total, nuclear and cytoplasmic NFATc3 bands from Western blotting by gray value. RASF and RASF-ASIC1a transfectants treated with pH 6.0 for 6 h. **(I)** Semi-quantitative analysis of total, nuclear and cytoplasmic NFATc3 bands from Western blotting by gray value. RASF and ASIC1a shRNA transfectants treated with pH 6.0 for 6 h. **(J)** Semi-quantitative analysis of nuclear NFATc3 bands from Western blotting by gray value. RASF treated with 1 mM EGTA, 10 μM BAPTA-AM, 5 μM verapamil and HMA. **(K)** Semi-quantitative analysis of total, nuclear and cytoplasmic NFATc3 bands from Western blotting by gray value. RASF treated with 100 nM PcTx-1.

Table S1. Primers for gene overexpression and shRNAs for gene silencing.

Gene		Primer Sequences
ASIC1a	Forward	5´-GAGGATCCCCGGGTACCGGTCGCCACCATGGAACTGAAGGCCGAGGAGGAG-3´
	Reverse	5´-TCCTTGTAGTCCATACCGCAGGTAAAGTCCTCGAACGTG-3´
Gene		shRNA
ASIC1a	shRNA1	CCGGCTATGGAAAGTGCTACACGTTCTCGAGAACGTGTAGCACTTTCCATAGTTTTT
ASIC1a	shRNA2	CCGGCGAGGTCATTAAGCACAAGCTCTCGAGAGCTTGTGCTTAATGACCTCGTTTTT
ASIC1a	shRNA3	CCGGGCTGTAGGCTACATCCTGATACTCGAGTATCAGGATGTAGCCTACAGCTTTTT
NFATc3	shRNA1	CCGGCGTCTCAGTTACAACCTATTACTCGAGTAATAGGTTGTAACTGAGACGTTTTT
NFATc3	shRNA2	CCGGGCCCAAGTATTCAAATTACATCTCGAGATGTAATTTGAATACTTGGGCTTTTT
NFATc3	shRNA3	CCGGGCCAGATGATTGTGCATCCATCTCGAGATGGATGCACAATCATCTGGCTTTTT

shRNA: short hairpin RNA; ASIC1a: acid sensing ion channel 1a; NFATc3: Nuclear factor of activated T-cells, cytoplasmic 3.

Table S2. Distribution of fluorescence arrays of 40 inflammatory cytokines.

POS 1	POS 2	POS 3	NEG	NEG	EOTAXIN-1	EOTAXIN-2	GCSF	GM-CSF	ICAM-1	IFN-g	I-309	IL-1a
POS 1	POS 2	POS 3	NEG	NEG	EOTAXIN-1	EOTAXIN-2	GCSF	GM-CSF	ICAM-1	IFN-g	I-309	IL-1a
IL-1b	IL-2	IL-3	IL-4	IL-6	IL-6sR	IL-7	IL-8	IL-10	IL-11	IL-12 p40	IL-12 p70	IL-13
IL-1b	IL-2	IL-3	IL-4	IL-6	IL-6sR	IL-7	IL-8	IL-10	IL-11	IL-12 p40	IL-12 p70	IL-13
IL-15	IL-16	IL-17	IP-10	MCP-1	MCP-2	M-CSF	MIG	MIP-1a	MIP-1b	MIP-1d	RANTES	TGF-b1
IL-15	IL-16	IL-17	IP-10	MCP-1	MCP-2	M-CSF	MIG	MIP-1a	MIP-1b	MIP-1d	RANTES	TGF-b1
TNF-a	TNF-b	sTNF RI	sTNF RII	PDGF-BB	TIMP-2	NEG	NEG	NEG	NEG	NEG	NEG	NEG
TNF-a	TNF-b	sTNF RI	sTNF RII	PDGF-BB	TIMP-2	NEG	NEG	NEG	NEG	NEG	NEG	NEG

POS: positive control; NEG: negative control; CCL:Chemokine (C-C motif) ligand; GCSF: granulocyte colony-stimulating factor; GM-CSF: granulocyte macrophage colony stimulating factor; ICAM-1: intercellular cell adhesion molecule-1; IFN-g: interferon gamma; IL: interleukin; CXCL: chemokine (C-X-C motif) ligand; MCP: monocyte chemoattractant protein; MIP: macrophage inflammatory protein; RANTES: regulated upon activation, normal T cell expressed and secreted; TGF: transforming growth factor; TNF: tumor necrosis factor; sTNF R: soluble tumor necrosis factor receptor; PDGF-BB: platelet-derived growth factor; TIMP-2: tissue inhibitor of metalloproteinases 2; MIG: monokine induced by gamma interferon; IP-10: interferon gamma-induced protein 10; IL-6 R: interleukin-6 receptor.

Table S3. Primers for ChIP-qPCR assay and RANTES promoter sequence for dual-luciferase reporter assay.

RANTES genomic sequence (3500 bp promoter+144 bp exon1+500 bp intron1)
AACAAATACGAATACATATTGACTGGGGGTTTAGTTTAGACTGTTGAAAAGCATAAATTACAGAATATC<br>CTTAAAGGCACTGACATCTTACTCTTAATGATAACATGATAATAATAGGCAATGTTATTAAGTACTTAACA<br>TGCGTCAGACGTTGCACTAAGAGCTTTGCAAGCATGAGAACATTTATAGGGACTTAATTGTCTCGATA<br>GTTGTGTGAGGAACTATTATTATTATTATCACTGATTTATAGGTAA***GGAAA***CTGAGACCAGAGAGATTA<br>TTAATAATTTACCTGAGGCCACCTGGACAATAAACAGGAGAAACTGCCGAGTCCCTGCTCTTAACCC<br>ACCGCCT***TTTCC***AGAACCTCTCACATGTTTGGTAACTTACCTTAGACCTAACGGGGCTTTGTGAA***GG***<br>***AAA***CAATTGGTAGAACATTTTTGTGTGGTTGGATGAATGTTTCTAAAGTGCTTGCAATTTGTTTCTTTA<br>AATGAGATGGCCATAC***TTTCC***AAAATTACG***TTTCC***ATTGTTAATTGATTGAGCTGTAAGGAGGGTATGA<br>GAGGATTGTTGTAAAGAGCTTGGAACAAGGCCAGGCGCAGTGGCTCGCATCTGTAATCCCAGCACT<br>TTGGGAGGCCGAGGTGGGCGGATCGCCTGAGGTCAGGAGTTCGAGACCAGCCTGGCCAACATGG<br>CGAAATCCTGTCTCTACTAAAAATACAAAAATTAGCCAGGCATGGTGGCGGACGCTTGTAATCCCAG<br>CTACTCAGGAGGCAGAAGAATTGCTTGAACCTGGGAGGTGGAGGGTACAGTGAGCCGAGATCGTG<br>CCACTGCACTTCAGCCTGATCAAAAAAGTGACACTCTGTCTCAAAAAAAAAAAAAAAAAAAAAGCTT<br>GGAATGGCACCTGTAACCTAGAAAGGTCTTTTTTTTTTTTTTTTTTTTTTTTGAGACGGAGTCTTGCTC<br>TTTCGCCCAGGCTGGAGTGCAGTGGCGCGATCTCAGCTCACTGCAAGCTCCGCCTCCTGGGTTCA<br>CGCCATTCTCCTGCCTCAACCTCTCGAGTAGCTGGGACTACAGGCGCCCGCCACCACGCCCGGCT<br>AATTTTTTCTATTTTTAGTAGAGACGGGGTTTCACCGTGTTAGCTAGGATGGTCTCGATCTCCTGACC<br>TCGTGATCCGCCCGCCTCGGCCTCTCAAAGTCCTGGGATTACAGGTGTGAGCCACCGCGCCTGGC<br>CGAAAGGTCTTGATAACTGTTAACTGACTTACTGATGTGATTCACTGGACAGAGCTTGAGCATGGTC<br>ATCAGAAACACCTGCCTTCAAATCCCACTTATCAGCTGCGTGATCTTGAAGATGTTAATTATCTGTTCT<br>GAGGCTTAC***TTTCC***TCTTTGGTCAAATGAGAAGAACAACCACTCTGCCTCAAAGTCATCGTGAGGAA<br>GAAATGTGGCAAAGTCTTCCCATTCTGGGTGATTTAGTCACCGTCAACCACCATGTTACAAGTGTGA<br>GAGTAGCTAACAGAAAATGATCTCAGTAGGGGGTTACATTTTGTTTATTTTGCTTTTGTCACTCTCTTT<br>TTTTTTTTTTTTTTTTTTTTGAGACGGAGTTTCGCTCTGTCGCCCAGGCTGGAGTGCAGTGGCGCGATC<br>TCGGCTCACTGCAAGCTCCACCTCCCGGGTTCACGCCATTCTCCTGCCTCAGCCTCCTGAGTAGCT<br>GGGACTACAGGCGCCCGCCACCACGCCCGGCTAATTTTTTGTATTTTTAGTAGAGGCGGGGTTTCA<br>CTGTGTTAGCCAGGATGGCCTCGATCTCCTGACCTCATGATCCGCCCGCCTCGGCCTCCCAAAGTG<br>CTGGGATTACAGGCGTGAACCACCGCGCCCGGCCGCTTTTGTCACTCTCAATAAACACTTTTCTCTT<br>AAATAATTCAGATGCCGGCCGGGTACGGCCTGTAATCCCAGCATTTTGGGAAGCCAAGGCGGGCGG<br>ATCACGAGGGCAGGAGATAGAGACCATCCTGGCTAACATGGTGAAACTCCATTTCTACTAAAAATACA<br>AAAAAATTAGCCAGGCATTCTGGCCAGCTCCTGTAGTCCCAGCTACTTGGGAGGCTGAGGCAGGAG<br>AATGGCGTGAACCTGGGAGGCACAGCTTGCAATGAGCCGAGATTGTGCCACTGCAGTCCAACCTG<br>GGCGACACAGCAAGACTGTCTCAATAATAATAATAATAATAATATTATTATTATTATTCAGATGCCAA<br>TTTCAGCCTAGCCCGGACCAAATTGTTGCTAATTCTGAACTACTAAAATAGGAATTGATGAAGACTTA<br>CTGTATCCAGTTCTTTGGAATGCAGTAAGGACCCTAGCTCTAATCCAGAAGTTCCACATGGGTTCAG<br>TTTTGAGGATACTCCTAACCACGAACCCTTTAATGTTGGGCAGTACTTAATGGACAGAGCACTGAAGT<br>GGGAGTGAGGGCAACTGGGTTCTGATCCCAACTCTGTCATTCATTTGCTGTGTGTTCTTAGCAAGTC<br>ACTCCTGCTCACTGGGCCTTGG***TTTCC***TCTTGTGCAACTGAAGAA**CAT*GGAAA*AA**ATGATCCCTAAA<br>GTCCTTTGAAGCTTTCATATTCTGTAACTTTTGTGCCCAAGAAGGCCTTACAGTGAGATGGGATCCCA<br>GTATTTATTGAG***TTTCC***TCATTCATAAAATGGGGATAATAATAGTAAATGAGTTGACACGCGCTAGGAC

AGTGGAATAGTGGCTGGCACAGATAAGCCCTCGGTAAATGGTAGCCAATAATGATAGAGTATGCTGTA
AGATATCTTTCTCTCCCTCTGCTTCTCAACAAGTCTCTAATCAATTATTCCACTTTATAAACAA***GGAAA***T
AGAACTCAAAGACATTAAGCACT***TTTCC***CAAAGGTCGCTTAGCAAGTAAATGGGAGAGACCCTATGA
CCAGGATGAAAGCAAGAAATTCCCACAAGAGGACTCATTCCAACTCATATCTTGTGAAAAGGTTCCC
AATGCCCAGCTCAGATCAACTGCCTCAATTTACAGTGTGAGTGTGCTCACCTCCTTTGGGGACTGTA
TATCCAGAGGACCCTCCTCAATAAAACACTTTATAAATAACATCCTTCCATGGATGAG***GGAAA***GGAGG
TAAGATCTGTAATGAATAAGCAGGAACTTTGAAGACTCAGTGACTCAGTGAGTAATAAAGACTCAGTG
ACTTCTGATCCTGTCCTAACTGCCACTCCTTGTTGTCCCCAAGAAAGCGGCTTCCTGCTCTCTGAGG
AGGACCCCTTCCCTGGAAGGTAAAACTAAGGATGTCAGCAGAGAAATT***TTTCC***ACCATTGGTGCTTG
GTCAAAGA***GGAAA***CTGATGAGCTCACTCTAGATGAGAGAGCAGTGAGGGAGAGACAGAGACTCGA
A***TTTCC***GGAGGCTATTTCAGTTTTCT***TTTCC***GTTTTGTGCAATTTCACTTATGATACCGGCCAATGCTT
GGTTGCTATTTT***GGAAA***CTCCCCTTAGGGGATGCCCCTCAACTGGCCCTATAAAGGGCCAGCCTGA
GCTGCAGAGGATTCCTGCAGAGGATCAAGACAGCACGTGGACCTCGCACAGCCTCTCCCACAGGT
ACCATGAAGGTCTCCGCGGCAGCCCTCGCTGTCATCCTCATTGCTACTGCCCTCTGCGCTCCTGCA
TCTGCCTCCCCATGTAAGTCCTGGTCTTGACCACCACAGCCCCTGGAGTCAGACTCTGCCTAGAAT
GCCTACCCCATCCCAATGACTGTCCCACACCTTGCCCTCTTTTCAACTGTAGCATGAATACTGTCTG
AACCCCATTATTTACATTCTTATGAATTGTCTTACCCATATGTCCTGTTGTCCTTGAGAGATCTCTATGC
TGCTTCATGGCAGGGATCTCCTGATCAGTTTTTCTGTCTTTAAGGTCTACACCCTCAAGGCCTACAG
GTGTTCACTGAGTGTGGACTCATGTTGGGGAGATTAGTAGTCATGAGTAGCTCACACTGACTTTGAA
ACACGTCTGACTCATGCCTGTCAGCAGCAGGGTTTCTAAGAAATTTAACTAAACTAAAACTTCCTGAG
ACCCTGAGACAGCCATATTCTCTCTCATTTCATAAATGTAAAAAAAATTGGCTTATGAGAGGGTATGTG
GCTTTATTAGAGTGAATACATTTAAAAAACAAATAAAAA

Primer1	Forward	5´-CAGAACCTCTCACATGTTTG-3´
	Reverse	5´-TTGGAAAGTATGGCCATCTC-3´
Primer2	Forward	5´-TCTTAGCAAGTCACTCCTGC-3´
	Reverse	5´-GATCCCATCTCACTGTAAGG-3´
Primer3	Forward	5´-TTGGTGCTTGGTCAAAGAGG-3´
Primer3	Reverse	5´-AATAGCAACCAAGCATTGGC-3´

RANTES promoter sequence (1812 bp)

GCTAGCTAATTTTTTGTATTTTTAGTAGAGGCGGGGTTTCACTGTGTTAGCCAGGATGGCCTCGATCT
CCTGACCTCATGATCCGCCCGCCTCGGCCTCCCAAAGTGCTGGGATTACAGGCGTGAACCACCGC
GCCCGGCCGCTTTTGTCACTCTCAATAAACACTTTTCTCTTAAATAATTCAGATGCCGGCCGGGTAC
GGCCTGTAATCCCAGCATTTTGGGAAGCCAAGGCGGGCGGATCACGAGGGCAGGAGATAGAGACC
ATCCTGGCTAACATGGTGAAACTCCATTTCTACTAAAAATACAAAAAAATTAGCCAGGCATTCTGGCC
AGCTCCTGTAGTCCCAGCTACTTGGGAGGCTGAGGCAGGAGAATGGCGTGAACCTGGGAGGCACA
GCTTGCAATGAGCCGAGATTGTGCCACTGCAGTCCAACCTGGGCGACACAGCAAGACTGTCTCAAT
AATAATAATAATAATAATATTATTATTATTATTCAGATGCCAATTTCAGCCTAGCCCGGACCAAATTGT
TGCTAATTCTGAACTACTAAAATAGGAATTGATGAAGACTTACTGTATCCAGTTCTTTGGAATGCAGTA
AGGACCCTAGCTCTAATCCAGAAGTTCCACATGGGTTCAGTTTTGAGGATACTCCTAACCACGAACC
CTTTAATGTTGGGCAGTACTTAATGGACAGAGCACTGAAGTGGGAGTGAGGGCAACTGGGTTCTGA
TCCCAACTCTGTCATTCATTTGCTGTGTGTTCTTAGCAAGTCACTCCTGCTCACTGGGCCTTGGTTT
CCTCTTGTGCAACTGAAGAA**CATGGAAAAA**ATGATCCCTAAAGTCCTTTGAAGCTTTCATATTCTGTA
ACTTTTGTGCCCAAGAAGGCCTTACAGTGAGATGGGATCCCAGTATTTATTGAGTTTCCTCATTCATA
AAATGGGGATAATAATAGTAAATGAGTTGACACGCGCTAGGACAGTGGAATAGTGGCTGGCACAGAT

AAGCCCTCGGTAAATGGTAGCCAATAATGATAGAGTATGCTGTAAGATATCTTTCTCTCCCTCTGCTTC
TCAACAAGTCTCTAATCAATTATTCCACTTTATAAACAAGGAAATAGAACTCAAAGACATTAAGCACTTT
TCCCAAAGGTCGCTTAGCAAGTAAATGGGAGAGACCCTATGACCAGGATGAAAGCAAGAAATTCCC
ACAAGAGGACTCATTCCAACTCATATCTTGTGAAAAGGTTCCCAATGCCCAGCTCAGATCAACTGCC
TCAATTTACAGTGTGAGTGTGCTCACCTCCTTTGGGGACTGTATATCCAGAGGACCCTCCTCAATAAA
ACACTTTATAAATAACATCCTTCCATGGATGAGGGAAAGGAGGTAAGATCTGTAATGAATAAGCAGGA
ACTTTGAAGACTCAGTGACTCAGTGAGTAATAAAGACTCAGTGACTTCTGATCCTGTCCTAACTGCC
ACTCCTTGTTGTCCCCAAGAAAGCGGCTTCCTGCTCTCTGAGGAGGACCCCTTCCCTGGAAGGTAA
AACTAAGGATGTCAGCAGAGAAATTTTTCCACCATTGGTGCTTGGTCAAAGAGGAAACTGATGAGCT
CACTCTAGATGAGAGAGCAGTGAGGGAGAGACAGAGACTCGAATTTCCGGAGGCTATTTCAGTTTT
CTTTTCCGTTTTGTGCAATTTCACTTATGATACCGGCCAATGCTTGGTTGCTATTTTGGAAACTCCCCT
TAGGGGATGCCCCTCAACTGGCCCTATAAAGGGCCAGCCTGAGCTGCAGAGGATTAAGCTT

NFATc3 binding site: 5´-**CATGGAAAAA-3´**

3´-GTACCTTTTT-5´

ChIP-qPCR: Chromatin immunoprecipitation-quantitative real-time PCR; RANTES: Regulated upon activation, normal T cell expressed and secreted.

Table S4. The expression of 40 inflammatory cytokines were examined by an inflammatory cytokines antibody array.

Eotaxin-1 (CCL11)	Eotaxin-2 (CCL24)	GCSF	GM-CSF	ICAM-1 (CD54)
IFN-gamma	I-309 (CCL1)	IL-1 alpha	IL-1 beta	IL-2
IL-3	IL-4	IL-6	IL-6 R	IL-7
IL-8 (CXCL8)	IL-10	IL-11	IL-12 p40	IL-12 p70
IL-13	IL-15	IL-16	IL-17A	IP-10 (CXCL10)
MCP-1 (CCL2)	MCP-2 (CCL8)	M-CSF	MIG (CXCL9)	MIP-1 alpha (CCL3)
MIP-1 beta (CCL4)	MIP-1 delta (CCL15)	RANTES (CCL5)	TGF beta 1	TNF alpha
TNF beta	sTNF RI	sTNF RII	PDGF-BB	TIMP-2

CCL: Chemokine (C-C motif) ligand; GCSF: granulocyte colony-stimulating factor; GM-CSF: granulocyte macrophage colony stimulating factor; ICAM-1: intercellular cell adhesion molecule-1; IFN-gamma: interferon gamma; IL: interleukin; CXCL: chemokine (C-X-C motif) ligand; MCP: monocyte chemoattractant protein; MIP: macrophage inflammatory protein; RANTES: regulated upon activation, normal T cell expressed and secreted; TGF: transforming growth factor; TNF: tumor necrosis factor; sTNF R: soluble tumor necrosis factor receptor; PDGF-BB: platelet-derived growth factor; TIMP-2: tissue inhibitor of metalloproteinases 2; MIG: monokine induced by gamma interferon; IP-10: interferon gamma-induced protein 10; IL-6 R: interleukin-6 receptor.

Table S5. The binding sites for NFATc3 in the *RANTES* gene promoter were predicted using the JASPAR database.

Matrix ID	Name	Score	Relative score	Sequence ID	Start	End	Strand	Predicted sequence
MA0625.1	NFATC3	11.9796	0.963359549	CCL5	2533	2542	-	TTTTTCCATG
MA0625.1	NFATC3	9.88731	0.910201022	CCL5	495	504	+	ACTTTCCAAA
MA0625.1	NFATC3	9.88619	0.910172431	CCL5	509	518	+	CGTTTCCATT
MA0625.1	NFATC3	9.71347	0.905784199	CCL5	2804	2813	+	TTATTCCACT
MA0625.1	NFATC3	8.78424	0.882175648	CCL5	350	359	+	CTTTTCCAGA
MA0625.1	NFATC3	8.37427	0.871759732	CCL5	1354	1363	+	ACTTTCCTCT
MA0625.1	NFATC3	8.20001	0.867332199	CCL5	252	261	-	AGTTTCCTTA
MA0625.1	NFATC3	7.90472	0.859829911	CCL5	407	416	-	TGTTTCCTTC
MA0625.1	NFATC3	7.89498	0.859582647	CCL5	2692	2701	-	CTATTCCACT
MA0625.1	NFATC3	7.71715	0.855064568	CCL5	2821	2830	-	TATTTCCTTG
MA0625.1	NFATC3	7.39961	0.846996784	CCL5	2633	2642	+	AGTTTCCTCA
MA0625.1	NFATC3	7.39776	0.84694996	CCL5	1081	1090	+	TTTTTCTATT
MA0625.1	NFATC3	7.39676	0.846924446	CCL5	568	577	-	TTGTTCCAAG
MA0625.1	NFATC3	7.12387	0.839991228	CCL5	2334	2343	+	AAGTTCCACA
MA0625.1	NFATC3	6.98871	0.836557329	CCL5	1489	1498	-	ATTTTCTGTT
MA0625.1	NFATC3	6.95327	0.835656906	CCL5	2931	2940	+	TCATTCCAAC
MA0625.1	NFATC3	6.82972	0.832517749	CCL5	1428	1437	+	TCTTCCCATT
MA0625.1	NFATC3	6.72564	0.829873567	CCL5	2508	2517	+	GGTTTCCTCT
MA0625.1	NFATC3	5.98541	0.811066825	CCL5	2852	2861	+	TTTTCCCAAA
MA0625.1	NFATC3	5.70279	0.803886464	CCL5	2299	2308	-	GCATTCCAAA
MA0625.1	NFATC3	5.56741	0.800446822	CCL5	2851	2860	+	CTTTTCCCAA
MA0625.1	NFATC3	5.56249	0.800321785	CCL5	2401	2410	-	TCTGTCCATT

NFATC3: also known as NFAT4, Nuclear factor of activated T-cells, cytoplasmic 3; CCL5: also known as RANTES, chemokine C-C motif ligand 5.
